# The quest for the identity of *Orthoceratiumlacustre* (Scopoli, 1763) reveals centuries of misidentifications (Diptera, Dolichopodidae)

**DOI:** 10.3897/zookeys.782.26329

**Published:** 2018-08-16

**Authors:** Marc Pollet, Andreas Stark

**Affiliations:** 1 Research Team Species Diversity (SPECDIV), Research Institute for Nature and Forest (INBO), Herman Teirlinckgebouw, Havenlaan 88 bus 73, B-1000 Brussels, Belgium Research Institute for Nature and Forest Brussels Belgium; 2 Research Group Terrestrial Ecology (TEREC), University of Ghent (UGent), K.L. Ledeganckstraat 35, B-9000 Ghent, Belgium University of Ghent Ghent Belgium; 3 Entomology Unit, Royal Belgian Institute of Natural Sciences (RBINS), Vautierstraat 29, B-1000 Brussels, Belgium Royal Belgian Institute of Natural Sciences Brussels Belgium; 4 Seebener Straße 190, 06114 Halle/Saale, Germany (also freelance collaborator at Senckenberg German Entomological Institute Müncheberg, Germany) Unaffiliated Halle/Saale Germany

**Keywords:** distribution, Dolichopodidae, ecology, Europe, North Africa, Mediterranean basin, *
Orthoceratium lacustre
*, *
Orthoceratium sabulosum
*, taxonomy, types

## Abstract

Recently, a species of *Orthoceratium* was collected in Greece that differs morphologically from the European species commonly presumed to be *Orthoceratiumlacustre* (Scopoli, 1763). Verification of the identity of the Greek species through comparison with 460 specimens of *Orthoceratium* from 17 West Palaearctic and one Afrotropical country, and examination of existing type material, revealed that the species recognized as *O.lacustre* in northwestern Europe for over 250 years is actually *O.sabulosum* (Becker, 1907), the other known species in the genus, which was originally described from Tunisia. Although the types of *O.lacustre* have been lost, a comparison of the distribution ranges of both species in Europe provided evidence that the species collected in Greece is conspecific with *O.lacustre*. Both species have distinct distributions in the West Palaearctic, with *O.lacustre* largely restricted to the northern border of the Mediterranean basin, and *O.sabulosum* more widespread, occurring in northwestern Europe, the western, southern, and eastern Mediterranean, the Middle East, and the Afrotropical Region (Tanzania). Both species are redescribed and fully illustrated, a neotype is designated for *O.lacustre* and a lectotype for *O.sabulosum*, and a key to males and females is provided. The misidentifications that lasted for over two centuries are explained by the omission by previous authors to study the type specimens, and inaccuracies in species descriptions and keys.

## Introduction

*Orthoceratium* Schrank, 1803 is a nearly exclusively West Palaearctic dolichopodid genus in the subfamily Hydrophorinae (Parent 1938), with only two known species (Yang et al. 2006). *Orthoceratiumlacustre* (Scopoli, 1763) has also been reported from Tanzania (Grichanov 1997, Grichanov and Brooks 2017), though the presence of this genus in subsaharan Africa has been considered doubtful (see Pollet et al. 2017). It seems most closely related to *Liancalus* Loew, 1857 and both differ from the other Palaearctic Hydrophorinae by uniseriate acrostichal bristles and a fore femur lacking ventral spines. The main features that separate *Orthoceratium* from *Liancalus* (based on the examination of two species in each genus) are given in the following key:

**Table:** 

1	Seven dorsocentral bristles. Two large inner and two smaller outer scutellar bristles. Two basal postpronotal bristles. One proepisternal bristle. Proepimeron simple. Male: fore femur with posteroventral pollinose spot. Fore tarsus simple. Abdomen with 5^th^ tergite with lateroventral process. Fore tarsus with one claw. Hypopygium with robust, large cercus	***Orthoceratium* Schrank, 1803**
-	Six dorsocentral bristles. Six equally strong scutellar bristles. One basal postpronotal bristle. Proepisternal bristles absent. Proepimeron with distinct ventral acute process. Male: fore femur simple. Fore tarsus with 2^nd^ tarsomere flattened. Abdomen with 5^th^ tergite simple. Fore tarsus with two claws. Hypopygium with small cercus with apical filiformous process	***Liancalus* Loew, 1857**

Pollet et al. (2017) reported on the recent and rather unexpected rediscovery of *O.lacustre* in Flanders (northern Belgium) after an absence of nearly 40 years. The authors also provided a full account of the distribution records of this conspicuous species in the western Palaearctic realm (Europe, North Africa, Middle East), and remarked that another *Orthoceratium* species had been collected in a mountainous region in Greece (further referred to as ‘species B’). At present, this hydrophorine genus only includes one other species, *O.sabulosum* (Becker, 1907), thus far only recorded from Tunisia.

In the process of verifying the identity of ‘species B’, the depository and availability of the type specimens of both described species was checked. This revealed that the Scopoli types of *O.lacustre* had been lost (Lorenzo Munari, pers. comm.), and that the status of *O.sabulosum* type specimens in Becker’s collection could be questioned. Moreover, series of *Orthoceratium* specimens from different European museums contained both *O.sabulosum* and ‘species B’. It thus appeared crucial to establish whether ‘species B’ actually corresponded with *O.lacustre* or represented a new, third species.

In the present paper, we present the results of this study, and give (re)descriptions of the species and information on their distribution and ecology. We finally discuss the plausible reasons for the continuous series of misidentifications, and the significance of type specimen examination.

## Materials and methods

Two specimens of *Orthoceratiumsabulosum* from the Becker collection (Museum für Naturkunde, ZMHB, Berlin, Germany) with a lectotype and paralectotype label resp., were examined. Although the validity of their designations might be questioned (see Redescription of *O.sabulosum*), there is no doubt that these specimens are part of Becker’s original type series. Types (presumably syntypes) of both *O.sabulosum* (initially deposited in the Hungarian Natural History Museum, HMHN, Budapest, Hungary) and *O.lacustre* (Scopoli collection) appear to be lost. All insects collected by Scopoli in Carniola were destroyed during fires in Scopoli’s house in Idria (Italy) in 1787 (Smith 1793, Roller and Haris 2008). None of these specimens could be located in the Museo Civico di Storia Naturale (MSNV, Verona, Italy) (Leonardo Latella, pers. comm.) or the Museo di Storia Naturale, Università degli Studi (MSNP, Pavia, Italy) (Carlo Giovanni Violani, pers. comm.). In addition, specimens determined as ‘*Orthoceratiumlacustre*’ from nine major European museums, one Turkish museum, one Bulgarian institute, the private collections of both authors (see further) and that of Miroslav Barták (Prague, Czech Republic) were also investigated.

(Re)descriptions are based on a large number of representative specimens of each species, both in alcohol and pin-mounted. A total of 173 character states was scored, with 35, 61, and 77 related to the head, thorax/abdomen/wing, and legs respectively. This allowed us to determine the most reliable and consistent decisive diagnostic features that were subsequently applied in the key.

Relevant non-genitalic diagnostic characters in collected specimens were photographed by the junior author. The hypopygium and tergite V of each species were drawn using a camera lucida. The left lateral view of the hypopygium is illustrated here. In describing the hypopygium, ‘dorsal’ and ‘ventral’ refers to the morphological position prior to genitalic rotation and flexion. Thus, in the drawings showing a lateral view of the hypopygium, the top is morphologically ventral, while the bottom is dorsal.

Biometrics were generally based on five specimens (wet = preserved in alcohol solution) of each gender in each of the two species unless otherwise mentioned, and include: (i) face width, (ii) body length, (iii) wing length (= distance between basis of basicosta and wing apex), (iv) relative wing width, (v) proximal versus apical section of vein M_1_, (vi) proximal versus apical section of vein CuA_1_, (vii) CuA_x_ ratio (= crossvein dm-cu versus apical section of vein M_1_) and (viii) relative lengths ratio of femur, tibia and tarsomeres of each leg. The latter relative lengths were recalculated so that the shortest leg part represents a value of “1”. Wing length was measured in both dry and wet specimens. All values given in this paper are average values, unless otherwise mentioned. Palp and proboscis size is compared to the eye size, measured as the vertical diameter (from about ocellar tubercle to the lower eye margin). Wing length was measured in 76 and 142 specimens of *O.lacustre* and *O.sabulosum* resp., to find out if differences occurred between both species and separate populations (see Table 1).

**Table 1.** Wing lengths (in mm) in males and females of *O.lacustre* and *O.sabulosum*. Measurements per country are given for those countries where at least five specimens of each sex were examined.

Biometrics	Mean (min–max)	No. specimens	Mean (min–max)	No. specimens
Sex	Male		Female	
* Orthoceratium lacustre *				
BULGARIA	5.8 (5.5–6.2)	9	6.3 (5.8–6.7)	11
FRANCE	5.7 (5.4–6.0)	13	6.1 (5.2–6.7)	13
ITALY	5.8 (5.4–6.2)	5	6.2 (5.7–6.5)	5
GREECE	5.8 (5.2–6.1)	7	6.4 (5.9–6.6)	8
All specimens	5.7 (5.2–6.2)	35	6.2 (5.2–6.7)	41
* Orthoceratium sabulosum *				
BELGIUM	5.6 (5.3–5.9)	20	6.1 (5.8–6.4)	20
Dudzele	5.6 (5.3–5.9)	5	6.1 (6–6.3)	5
Lissewege	5.8 (5.6–5.9)	5	6.2 (5.9–6.4)	5
Knokke (Het Zwin)	5.7 (5.5–5.9)	5	6.1 (5.8–6.3)	5
GREAT BRITAIN	5.5 (5.2–5.8)	19	5.9 (5.2–6.4)	17
NETHERLANDS	5.8 (5.5–6.0)	6	6.1 (5.7–6.5)	5
SPAIN	5.4 (4.5–5.8)	14	5.8 (5.5–6.1)	5
All specimens	5.6 (4.5–6.1)	77	6 (5.2–6.5)	65

**Figure 1.**
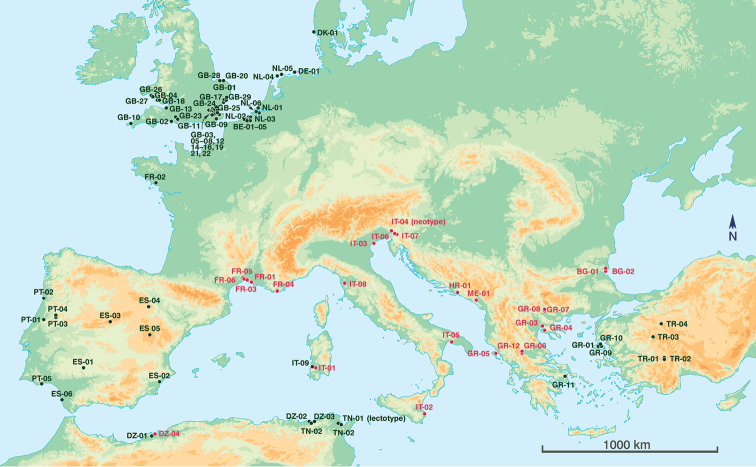
Distribution ranges of *Orthoceratiumlacustre* (red symbols, incl. type locality of neotype) and *O.sabulosum* (black symbols, incl. type locality of lectotype) in the West Palaearctic (Iranian records not included). Information related to the site codes is given in Suppl. material 1. List of (non-type) records of *Orthoceratium*.

Capture locations of *Orthoceratium* specimens are given in Figure 1, if sufficient information on the site was available either from the label of the specimen or – if this was lacking – as provided by the collection curator. Only specimens were considered which had been effectively examined, in most cases by the senior author. Each location has been positioned on the map as accurately as possible, based on the information available. If only the locality (e.g., a city) was known, then the symbol in Figure 1 is shown in the centre of this locality which might not necessarily correspond exactly with the actual collecting site.

The general morphological terminology follows Cumming and Wood (2009), while Brooks (2005) was used for male genitalia. The following abbreviations were used:

**ac** acrostichal bristles;

**ad** anterodorsal;

**ant pprn** anterior postpronotal (= humeral *sensu*Parent 1938);

**ap** apical;

**apv** apicoventral;

**av** anteroventral;

**bas pprn** basal postpronotal (= posthumeral *sensu*Parent 1938);

**bv** basoventral;

**dc** dorsocentral bristle pairs;

**ds** dorsal;

**MSSC(s)** male secondary sexual character(s);

**npl** notopleural;

**pal** postalar;

**pd** posterodorsal;

**psut ial** presutural intra-alar (= presutural *sensu*Parent 1938);

**pv** posteroventral;

**S** abdominal sternite;

**spal** supra-alar;

**sut ial** sutural intra-alar (= sutural *sensu*Parent 1938);

**ta** tarsomere, _1-5_ in the descriptions of tarsi refers to basal (_1_) to apical (_5_) tarsomeres;

**T** abdominal tergite;

**vt** ventral;

**I**, **II**, **III** refers to fore, mid and hind leg;

**I–VI** in the descriptions of abdominal segments (tergites/sternites) refers to basal (_I_) to caudal (_VI_) segments.

Institutional, collection and other abbreviations:

**ANSC** Andreas Stark private collection, Halle/S., Germany;

**NHMUK**The Natural History Museum, London, UK;

**HMNH**Hungarian Museum of Natural History, Budapest, Hungary;

**IBER** Institute of Biodiversity and Ecosystem Research, Sofia, Bulgaria;

**MAPC** Marc Pollet private collection, Welle, Belgium;

**MIBC** Miroslav Barták private collection, Prague, Czech Republic;

**MNHN**Muséum national de l’Histoire naturelle, Paris, France;

**MLUH** Zentralmagazin naturwissenschaftlichen Sammlungen, Martin-Luther-Universität, Halle/S., Germany;

**NHMW**Naturhistorisches Museum, Vienna, Austria;

**RBINS**Royal Belgian Institute of Natural Sciences, Brussels, Belgium;

**RMNH**Naturalis Biodiversity Centre, Leiden, Netherlands;

**ZFMK**Zoologisches Forschunginstitut und Museum A. Koenig, Bonn, Germany;

**ZLKU** Zoology Laboratory, Department of Biology, Faculty of Science, Muğla Sıtkı Koçman University, Muğla, Turkey;

**ZMHB**Museum für Naturkunde, Berlin, Germany;

**ZMUC**Zoological Museum, University of Copenhagen, Copenhagen, Denmark.

Other abbreviations: **MT**: Malaise trap, **SW**: collected by sweepnet.

Label information of mounted specimens is provided in full and with the original spelling. If not indicated otherwise, the label was white and rectangular, and information is from the top side. Label information is given from the top downward, with data from each label between quotation marks, and data from different lines on the same label separated by a slash (/). Information from different labels is separated by a semi-colon (;). The species record is followed by the repository of each specimen between square brackets []. In addition to the label information, for non-type specimens, the most relevant label information is enriched, uniformly structured and given in the following format: “(site code) – COUNTRY: ♂, ♀, province (or equivalent administrative division), locality, location/area, latitude, longitude, altitude, sampling date (start) – sampling date (end), sampling method, collector [collection]” (see Suppl. material 1. List of (non-type) records of *Orthoceratium*). The site code is also used in Figure 1. All specimens examined were pinned, unless otherwise mentioned (W: wet alcohol sample).

## Results

A total of 428 specimens of *Orthoceratium* from eight European museums and three other collections has been examined, mainly by the senior author; the identity of two, two, five and 23 additional specimens from HMNH, ZMUC, ZLKU and MIBC was kindly checked by Zoltán Soltész, Thomas Pape, Alper Tonguç, and Miroslav Barták, resp. Laurence Clemons (Kent, UK) also confirmed the identity of the specimens listed in Clemons (2003). The following museums did not hold any identified *Orthoceratium* material: NMPC: National Museum (Natural History), Prague, Czech Republic; MSNVE: Museo di Storia Naturale, Venice, Italy. The specimens originated from 17 different countries in the West Palaearctic, including 13 European, two North African and two Middle East ones, and one Afrotropical country.

### Literature study

To our surprise, the examination of the type material of *Orthoceratiumsabulosum* revealed that the species widely known (and collected) as ‘*O.lacustre*’ in northwestern Europe was conspecific with this species. This held true for nearly all specimens of ‘*O.lacustre*’ examined from North Africa and the Middle East (Turkey, Iran) as well. The question evidently raised if ‘species B’ then represented the true *O.lacustre* or not. As mentioned before, establishing the species concept of the latter species proved difficult due to the loss of the type material. Hence, the original description and other literature sources were studied carefully in search for information on significant diagnostic features that matched those of ‘species B’.

Scopoli (1763) described *Muscalacustris* (later transferred to *Orthoceratium*, most presumably by Schrank (1803)) as follows:

“Diagn. Thorax aeneus. Abdomen viridi-aeneum. Ambulat super aquas stagnantes tanquam *Cimex Lacustris* [now in *Gerris*]. Habitat in lacubus. Frons subargentea. Oculi virides. Pili duo divaricati in occipite. Antennae nigrae, clavatae, obtusae. Rostrum palpis subvillosis, parvis. Thorax aeneus, glaber. Alae hyalinae, immaculatae; costa antice ferruginea. Scutellum edentatum, rotundatum, pilosum. Abdomen lineam longum, viridi-aeneum, albido villo adspersum, subtus subfuscum: segmentis lateraliter punctatis. Pedes longi: lamellis unguium pallidis.”

Unfortunately, all listed characters fit both *O.sabulosum* and ‘species B’. Scopoli’s species, however, seemed to occur in stagnant water bodies (see original description) like e.g., inland lakes, and did not seem to be confined to saltmarshes or brackish marshes like *O.sabulosum* in northwestern Europe.

As the Scopoli type specimens of *O.lacustre* were destroyed as early as 1787, it is unlikely that Haliday (1851), Loew (1857), Schiner (1862) or Mik (1878) had the opportunity to examine these specimen(s). Haliday’s (1851: 182–183) description of this species only contains the following relevant information:

“Wings hyaline, usually tinged with ferruginous towards the fore edge, … Abdomen of the male … lamella oblong, compressed, broad at tip and truncated. … On waters, both fresh and brackish. (E[ngland]. I[reland]).”

However, it can be assumed that Haliday based this description on ‘*O.lacustre*’ from England or Ireland, which now appears to be *O.sabulosum*.

Schiner (1862) gives the following description (of *Liancaluslacustris*):

“Beine schwarz mit gelben Knieen und Gelenken. — Metallisch-grün. Untergesicht silberweiss schimmernd. Fühler schwarz. Rückenschild undeutlich gestriemt. Analanhänge länglich, zusammengedrückt, am Ende breit und abgestutzt. Schenkel oben grün, auch der hinterste Metatarsus. Flügel glashell, gegen den Vorderrand gewöhnlich bräunlich, gelblich tingirt, die vierte Längsader gebrochen. 2 2/3 ‘’’. Nach Scopoli in [Herzogtum] Krain; ich erhielt die Art durch Hrn. Micklitz aus dem Küstenlande.”

Neither Loew (1857) nor Mik (1878) provided useful information for the recognition of *O.lacustre*. The former author did mention that *O.lacustre* (then in *Liancalus*) was considered much rarer than *Liancalusvirens* (Scopoli, 1763) and that its distribution ranged from England to Sicily. It seems like he actually saw specimens as he reports on the colour of teneral specimens.

Becker (1907) described *Alloeoneurussabulosus* on the basis of specimens from Tunis (HMHN); two years later this genus was listed as synonym of *Orthoceratium* by Kertész (1909). Becker (1907) initially remarked that this species is different from the known and described species, larger than *A.lacustris* and featuring brownish wings. In his key to the species, he summarizes the most diagnostic features that separate both species as follows:

**Table:** 

“Face white, hardly wider than postpedicel (length) (male). Mesonotum dusted whitish grey on dorsum; sternite IV with ventral process (male). Wing entirely hyaline. Size 5 mm	*O.lacustre* (Scopoli, 1763)
Face grey, about 2 × as wide as postpedicel (length) (male). Mesonotum dusted yellowish grey on dorsum; sternite IV normal (without process) (male). Wing brownish. Size 6 mm	*O.sabulosum*” [trans.]

The only feature of *O.lacustre* in this key that matches ‘species B’ is the narrower face, compared to *O.sabulosum*. But specimens of both species show an equally large variation in the colour of the wings and the dusting of their pronotum. This is quite remarkable as we believe that Becker actually had specimens of both species at hand when he described *O.sabulosum*, as he repeatedly refers to *O.lacustre*. Why he used doubtful differences in his key or even mixed up features (see further) is unclear, but possibly he only had one single or a small number of *O.lacustre* specimens to compare with.

Ten years later, Becker (1917) published a similar key and added a drawing of the hypopygium and wing of *O.lacustre*. The shape of the ventral process of the 5^th^ tergite (not 4^th^ sternite, as mentioned by Becker 1907!) and the dense pubescence on the cercus apex clearly match those of ‘species B’.

Parent (1938) mentioned that *Orthoceratium* includes littoral species that occur at the borders of small streams and lakes in saltmarshes (‘*Slikke des Belges*’), and reported *O.lacustre* from France, and *O.sabulosum* from Tunisia. His description of *O.lacustre*, however, clearly points to *O.sabulosum*: face wide, about 2/5 of eye width, and a black bristle present amid coxa I. Some of his figures (fig. 466: postpedicel, fig. 467: abdomen, especially the posteroventral process of T_V_) confirm this conclusion.

The key to both species by Negrobov (1979) is largely a copy of the Becker (1907) key. He omitted the body length as a diagnostic feature and corrected the sternite IV into tergites IV–V. He also gives a full description of both species. Only drawings of the genitalia of *O.lacustre* are included (Negrobov 1978) though the author mentions to have examined the *O.sabulosum* types (ZMHB), however, without giving details on the specimens. On the basis of the description and especially the drawings of the hypopygium (figs 1331–1334, 1335, 1337), we can conclude that Negrobov’s *O.lacustre* matches ‘species B’ exactly. Next to the fact that it was unclear where the specimens – that he used for the description of *O.lacustre* – originated from (Oleg Negrobov, pers. comm.), the question remained if his species (and ‘species B’) was the true *O.lacustre*. Indeed, Negrobov was not able to examine the type specimens of *O.lacustre* nor did the extant literature provided decisive information (as shown above).

### Distribution patterns

In a second stage of the verification process the type locality was considered to comprise a possible clue about the identity of *O.lacustre*. Scopoli collected the species in Carniola (‘Krain’ in German), a historical region that corresponds mainly with inland parts of present-day Slovenia, including mountains. Since Scopoli stated that the species skated on backwaters [“*ambulat super aquas stagnantes*”] and occurred in or along lakes [“*in lacubus*”], it could further be assumed that he collected the species in inland wetland habitats (and not along the coast).

**Figure 2.**
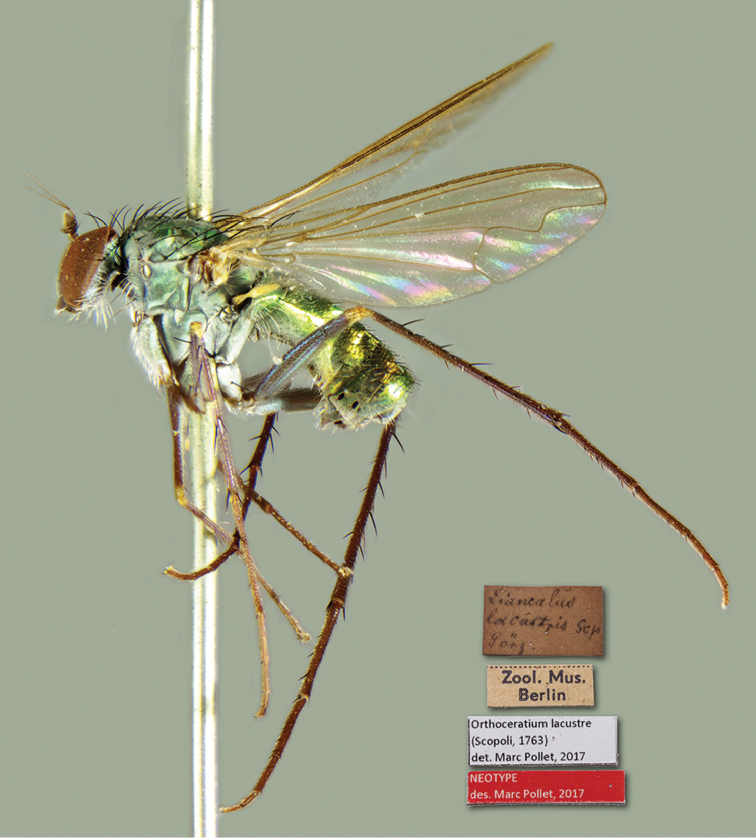
*Orthoceratiumlacustre*, habitus (male neotype), and the original labels.

To find out exactly where this type locality was situated within the distribution ranges of *O.sabulosum* and ‘species B’, capture locations of both species were plotted on a map of the West Palaearctic (see Figure 1). This clearly revealed that the ranges of both species only just overlap at three sites (Algeria, Greece, Sardinia) but otherwise show a distinctly different distribution: *O.sabulosum* seems to occupy the coastal region of northwestern Europe, western (Spain, Portugal), southwestern (Algeria and Tunisia in North Africa) and eastern borders of the Mediterranean basin (Greece, Turkey), but also Iran (not indicated on the map, see Kazerani et al. 2014) and Tanzania (not indicated on the map, Grichanov and Brooks 2017; see also further). In contrast, ‘species B’ seems to be largely restricted to the northern Mediterranean region. As the type locality of *O.lacustre* is situated within the distribution range of ‘species B’, and no other species has been detected in the extensive examined material, it could finally be concluded that ‘species B’ must be conspecific to *O.lacustre*. Subsequently, a male specimen collected in Görz [= Gorizia] (Italy), the locality closest to the type locality of this species, was selected as neotype (see below, Figure 2).

### Systematic accounts (see Pape et al. 2011)

Order Diptera Linnaeus, 1758

Suborder Brachycera Macquart, 1834

Clade Eremoneura Lameere, 1906

Superfamily Empidoidea Latreille, 1804

Family Dolichopodidae Latreille, 1804

Subfamily Hydrophorinae Becker, 1917

Genus *Orthoceratium* Schrank, 1803 (monotypic)

#### Orthoceratium lacustre

Taxon classificationAnimaliaDipteraDolichopodidae

(Scopoli, 1763)

Figs 1 , 2 , 3A–B , 4A, B, D, F , 5A–B , 6A–B , 7 , 10B

Musca lacustris Scopoli, 1763: 343. Type locality: Carniola (= present-day Slovenia) – presumably transferred to Orthoceratium by Schrank (1803)

##### Notes on synonyms.

*Muscaformosa* Haliday, 1832 and *Medeterusviridipes* Macquart, 1834, previously listed as synonyms of *O.lacustre*, clearly refer to *O.sabulosum* (see further).

##### Diagnosis.

**Figure 3.**
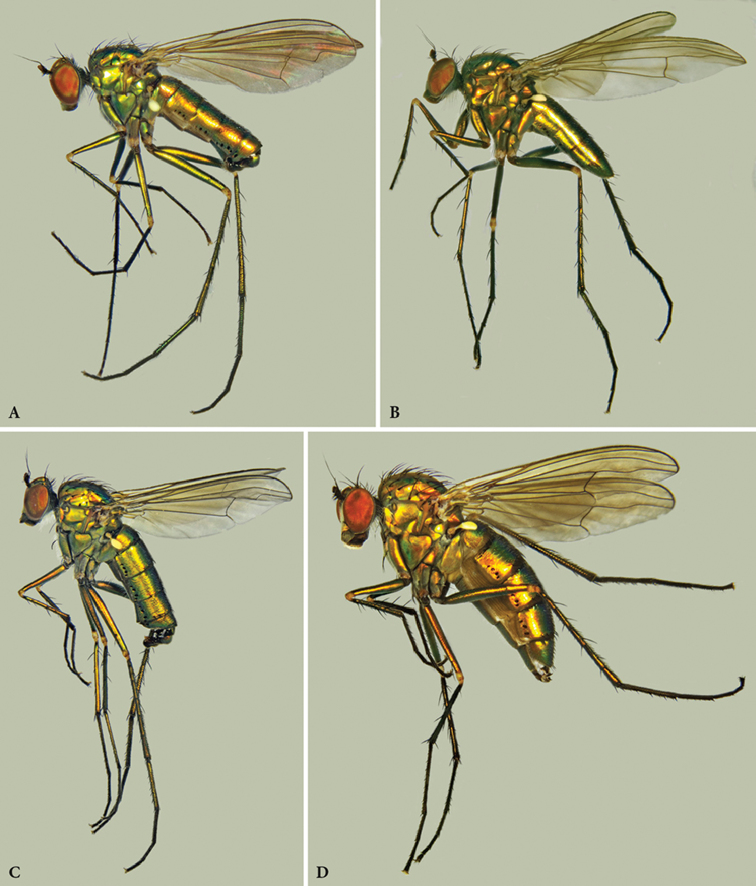
*Orthoceratiumlacustre*, habitus (W): **A** male **B** female. *Orthoceratiumsabulosum*, habitus: **C** male **D** female. Same scale in all pictures.

**Figure 4.**
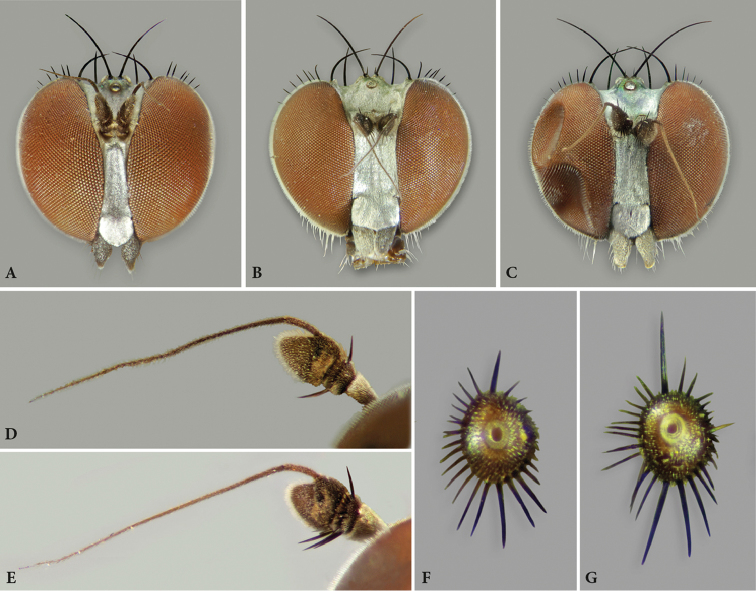
*Orthoceratiumlacustre*, head: **A** face (male) **B** face (female) **D** antenna (male) **F** pedicel, posterior view (male). *Orthoceratiumsabulosum*, head: **C** face (male) **E** antenna (male) **G** pedicel, posterior view (male).

**Figure 5.**
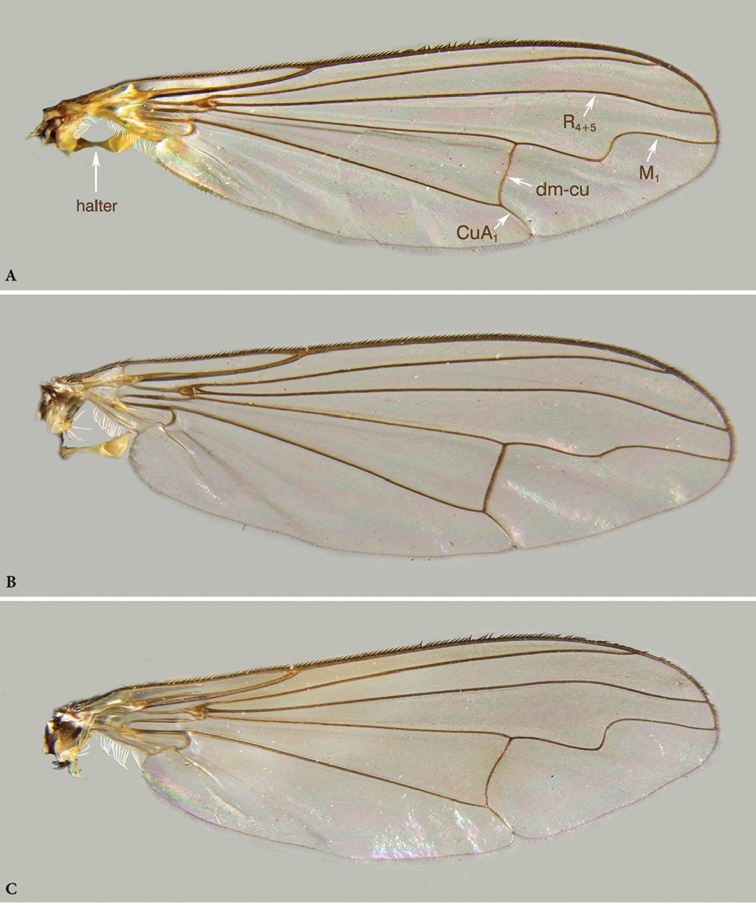
*Orthoceratiumlacustre*, wing and halter: **A** male **B** female. *Orthoceratiumsabulosum*: **C** wing (male). Veins R_4+5_, M_1_, CuA_1_, and dm-cu indicated.

**Figure 6.**
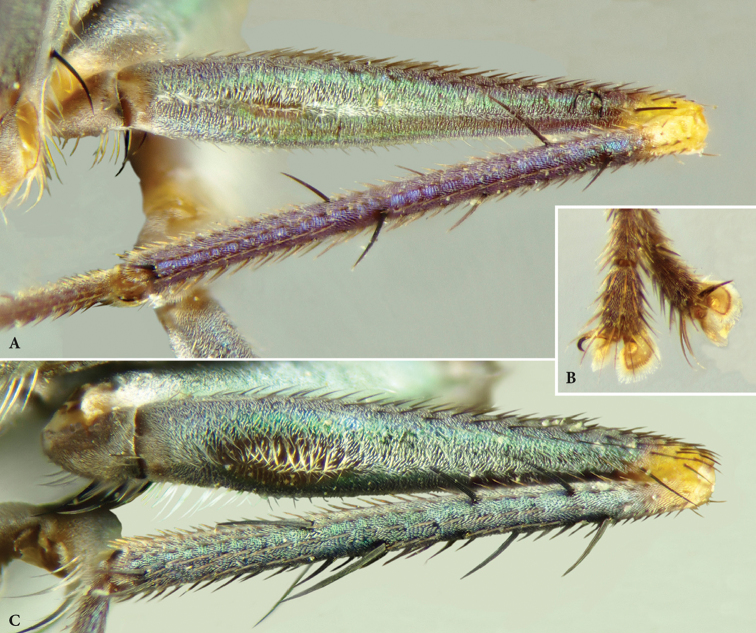
*Orthoceratiumlacustre*, male: **A** femur I (posteroventral view) **B** tarsomere I_5_, with left tarsus on the left and right tarsus on the right hand side (dorsal view). *Orthoceratiumsabulosum*, male: **C** femur I (posteroventral view).

Large, short-bodied, slender, entirely green species with abdomen 1.6 × as long as thorax (Figs 2, 3A, B). All legs mainly dark and metallic with narrowly yellow knees. Wing smokey reddish yellow, with reddish yellow veins (Fig. 5A, B). Apical section of vein M_1_ with strong sinous bend at ½. Posterior border of wing indented at vein CuA_1_. Coxa I with strong white pubescence, and with three black bristles only at apex. Coxa II with only pale bristles at apex anteriorly. Pedicel with short apical bristles (Figure 4F). Ac uniseriate, rather small, at most 1/3 as long as dc. Male: face not as wide as postpedicel is long (Figure 4A). Postpedicel elongate triangular, at least 1.2 × as long as deep (Figure 4D). T_V_ with blunt ventral process at each side bearing short dark separate bristles (Figure 10B). Femur I with small ovoid brownish yellow pv tuft just beyond basal 1/4, about 1/8 of femur length (Figure 6A). Femora I–II with multiple rows of very short white erect setae on basal ½. Tibia II with three ad bristles, with basal bristle shorter, and with one av bristle. Tibia III with four strong and one small pd bristles. Tarsus I with only one claw, and tarsomere taI_1_ mostly unmetallic (Figure 6B).

##### Redescription.

**Figure 7.**
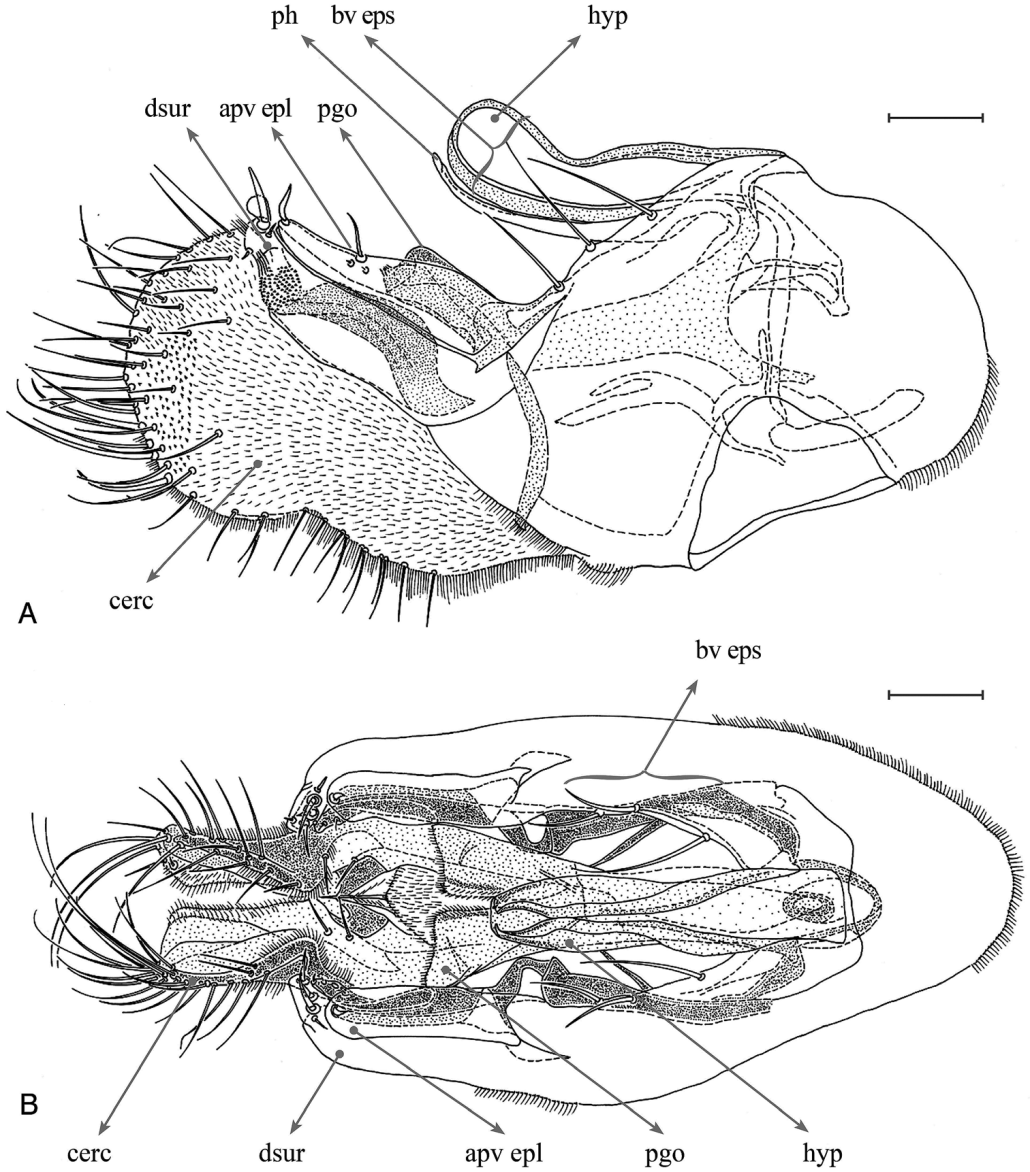
*Orthoceratiumlacustre*, hypopygium: **A** left lateral view **B** ventral view. Abbreviations: hyp: hypandrium, ph: phallus, bv eps: basoventral epandrial setae, apv epl: apicoventral epandrial lobe, dsur: dorsal surstylar lobe, pgo: postgonites, cerc: cercus. Scale bars: 0.1 mm.

**Male**. Body length: 5.0–5.7 mm (n = 25); wing length: 5.2–6.2 mm (n = 44), 0.3 × as wide as long. **Head** (Fig. 4A, B, D, F). Face silvery white, slightly narrowing towards middle of face, then widening towards clypeus, latter with triangular lower margin, weakly projecting; face 0.9 × as wide as postpedicel (length), with short white pubescence. Frons with metallic green ground colour, strongly dusted yellowish white. Occiput with metallic green ground colour, dusted whitish, convex in middle. Palp 1/5–1/4 of eye, triangular, dark brown, strongly dusted whitish, with white pubescence, and apical bristle absent. Proboscis dark brown. Eyes red, with short white pubescence. Uppermost seven to nine postocular bristles erect, black, and lower bristles curved, white, forming whiskers. One pair of black postocellar bristles. Antenna entirely dark brown, with scape bare and pedicel with apical crown of short bristles; postpedicel dark brown, elongate triangular, with blunt apex, 1.2–1.4 × as long as deep, 0.9–1.3 × as long as scape and pedicel combined, with short pubescence; arista-like stylus dorsal, inserted at middle of upper rim of postpedicel, 2.0–2.4 × as long as first three antennal segments combined, bare. **Thorax** (Figs 2, 3A). Mesonotum entirely brilliant metallic green with sometimes bluish violet tinge, strongly dusted greyish white on pleura and certain zones on dorsum, only without dusting between dc and ac, and between dc and npl areas; scutellum bluish violet, bare on dorsum, with four marginal bristles, lateral pair much smaller than median pair. Anterior spiracle with group of multiple curved, yellowish white, long setae. Thoracic bristles black. Seven dc, with 1^st^dc laterally off-set, and 6–7^th^dc stronger; three to five ac, uniseriate, reaching level between 5^th^ and 6^th^dc, rather small, at most 1/3 × as long as dc; with two strong black and one minute white ant pprn, one internal and one external bas pprn, one psut ial, one sut ial, two npl, two spal, and one pal bristles. Upper proepisternum with a large group of long yellowish white curved setae; lower proepisternum with one strong black curved bristle and a small group of yellowish white curved setae. **Wing** (Figure 5A). Smokey reddish yellow, with reddish yellow veins. Vein R_4+5_ sinuous near wing apex, there parallel with vein M_1_; apical section of vein M_1_ with strong sinous bend at ½ (MSSC); crossvein dm-cu rather straight; posterior border of wing indented at vein CuA_1_. Proximal section of vein M_1_ 1.9 × as long as apical section. Proximal section of vein CuA_1_ 8.5 × as long as apical section. CuA_x_ ratio: 1.7. Halter pale, calypteral fringe yellowish white. **Legs** (Figs 2, 3A, 6A–B). Overall dark, metallic green to violet, with pale yellow knees in all legs, and with black bristles. Coxae dark, with metallic green ground colour and strongly dusted whitish, coxae I–II with about apical 1/4 yellow, coxa III with about apical 1/3 yellow. Coxa I with dense, white pubescence and three rather small, black ap bristles. Coxa II with dense white pubescence on anterior face, and one black inclined bristle at 1/2 on margin; lateral face bare. Coxa III with one black, erect external bristle, inserted at 1/2, with vertical row of white setae. Trochanters dark brown. Femora I–III brilliant metallic green, sometimes with violet tinge, femora I–II with pale yellow knee on apical 1/8, and on apical 1/10 in femur III. Femur I with multiple rows of very short white erect setae on basal ½ (MSSC); with small ovoid brownish yellow pv tuft just beyond basal 1/4, about 1/8 of femur length (MSSC); with one rather small pv preapical bristle. Femur II with one strong ad preapical bristle, at less than apical 1/5, and with one small pv preapical bristle; with one row of very short white erect vt setae on basal 1/3 (MSSC), and with one row of short inclined pv setae along entire length, white on basal 2/3 and black on apical 1/3, longest at basis and apex. Femur III with one strong ad preapical bristle, at about apical 1/3, and one small pv preapical bristle; sometimes with some thin inclined (thus not erect!) ds bristles in basal 1/5. Tibiae I–III brilliant metallic green to violet, tibia I with basal 1/8, tibia II with basal 1/9, and tibia III with less than basal 1/10 pale yellow. Tibia I with two ds bristles, 2–3 × as long as tibia is deep; with two small ad bristles, 1–1.5 × as long as tibia is deep, and with two to three pv bristles, 2–3 × as long as tibia is deep; with white pilosity on av face along entire length, and with two small ap bristles. Tibia II with three ad bristles, about 3 × as long as tibia is deep, with basal bristle shorter; with two pd bristles, 2 × as long as tibia is deep, with basal bristle shorter, and with four ap bristles; with one av bristle at basal 2/3 and one pv bristle at basal 1/5, both 2 × as long as tibia is deep; and two small pv bristles in apical 1/2, not as long as tibia is deep. Tibia III with five ad bristles, about 2.5 × as long as tibia is deep, four strong and one small pd bristles, former about 2.5 x, latter not as long as tibia is deep, and four strong ap bristles; with distinct pd row on apical 1/2; with three-four av bristles, 1–1.5 × as long as tibia is deep, and with multiple shorter pv setae along entire length. Tarsi I–III dark brown, with taI_1_ mostly unmetallic and taII_1_ and taIII_1_ with metallic green to violet reflection. Tarsus I with taI_1_ with pale ventral pubescence (MSSC) with some darker short bristles; taI_5_ with long curved dorsal setae at apex, 0.7 × as long as taI_5_ length; only inner claw present (MSSC). Tarsus II with taII_1_ with multiple short vt bristles, nearly as long as taII_1_ is deep. Tarsus III with taIII_1_ with multiple inclined vt bristles, longest about as long as taIII_1_ is deep. Ratio of femur/tibia/tarsomeres 1–5 in leg I: 10.3/11.4/6.3/2.2/1.5/1/1.1, in leg II: 9.5/12.2/8.4/3.7/2.4/1.3/1, and in leg III: 10.6/15.3/6.7/4.6/2.8/1.4/1. **Abdomen** (Figure 2, 3A). Basal five segments pubescent, 6^th^ bare, minute, only visible dorsally. T brilliant metallic green, with bluish or with bronze tinge in some specimens, strongly dusted whitish on lower margins, with short dense black pubescence on dorsum, long yellowish white setae laterally, strongest on sides of T_I_, and T_I–IV_ with black bristles on posterior margin, strongest on T_I_; T_V_ brilliant metallic green without prominent dark bristles on posterior margin, with blunt ventral process at each side, with short dark separate bristles (MSSC) (Figure 10B). ST with green ground colour, strongly dusted whitish, with yellowish white, erect bristles. Hypopygium (Figure 7) with epandrium concolorous with tergites; hypandrium rather stout with subcircular apex, with ventrally curled up sides forming a gutter; phallus slender and strongly curved; three basoventral epandrial setae of subequal size, and apicoventral epandrial lobe pale reddish yellow, stout, narrowing towards apex and adjacent to outer surstylar lobe; surstylus with robust pale reddish yellow outer (or dorsal) lobe with area of minute spines near apex and with subcircular bristle at apex, inner (or ventral) surstylar lobe dark, robust with tapering apex baring a few bristles; postgonites robust, dark, with apical pubescence and a vt process; cercus dark brown, medium-sized, rather rectangular, apex large subcircular, with dense yellow pubescence, dark brown. **Female** (Figure 3B). Body length: 5.4–6.2 mm (n = 23); wing length: 5.2–6.7 mm (n = 51), 0.3 × as wide as long. As male, except for: abdomen 1.4 × as long as thorax, slender. Face 1.7–2.3 × as wide as postpedicel (length). Frons ground colour metallic green, strongly dusted yellowish white. Palp ovoid. Uppermost eight to ten postocular bristles black. Antenna dark brown, with scape sometimes paler (yellowish brown); postpedicel triangular, with blunt apex, 0.9–1.1 × as long as deep, 0.8–0.9 × as long as scape and pedicel combined; arista-like stylus 2.4–2.7 × as long as first three antennal segments combined. Thorax with four to five ac, reaching between 4^th^ or 5^th^dc. Wing (Figure 5B) with vein R_4+5_ bended but straight near wing apex, there parallel with vein M_1_; apical section of vein M_1_ with weak bend (sinuous) at 1/2; crossvein dm-cu straight. Proximal section of M 1.7 × as long as apical section. Proximal section of CuA_1_ 6.9 × as long as apical section. CuA_x_ ratio: 1.6. Coxa I with two to three rather small, black ap bristles. Femur I bare ventrally. Femur III often with some thin inclined ds bristles in basal 1/5. Tibiae I–II with basal 1/10 pale yellow; tibia I with three pv bristles. Tibia II with two av bristles at basal 2/5 and 2/3, 2–2.5 × as long as tibia is deep, and four small pv bristles on entirely length, not as long as tibia is deep. Tibia III with ad bristles about 3 × as long as tibia is deep, and with four strong pd bristles, 2.5–4 × as long as tibia is deep. Tibia III with av bristles 1.5–2.0 × as long as tibia is deep. Tarsus I with taI_1_ with multiple short black ventral bristles, nearly as long as taI_1_ is deep; taI_5_ with both claws. Ratio of femur/tibia/tarsomeres 1–5 in leg I: 8.6/9.5/5.7/2.3/1.5/1/1.1, in leg II: 9.1/11.6/8.1/3.4/2/1.2/1, and in leg III: 10.6/15/6.4/4.3/2.6/1.4/1. Abdomen with five pubescent segments, 6^th^ invisible; also ST_IV_ with strong whitish dusting.

##### Type specimens.

**ITALY**: **NEOTYPE** (here designated to fix the identity of the species) ♂, [brownish rectangular] “*Liancalus*/ *lacustris* Scp”/ “Görz.” [= Gorizia, in Friuli-Venezia Giulia region]; “Zool. Mus.”/ “Berlin”; “*Orthoceratium lacustre*”/ “(Scopoli, 1763)”/ “det. Marc Pollet, 2017”; [red rectangular] “NEOTYPE” / “des. Marc Pollet, 2018” (2017 on initial label in Figure 1) [ZMHB] (IT-04).

##### Other material examined.

See Suppl. material 1. List of (non-type) records of *Orthoceratium*.

##### Remarks.

In order to fix the identity of the species, a neotype of *O.lacustre* was selected on the basis of the locality (closest to the original type locality or region), and the preservation status of the specimen (see Figure 2).

Of all examined specimens (n = 131) one Trieste specimen featured a strong curved black bristle on the right fore coxa.

##### Distribution.

As a result of the taxonomic mix-up between both species in the past, previous distribution records of *O.lacustre* in the literature – many of which refer to *O.sabulosum* in reality – must be considered unreliable. Our present study revealed that *O.lacustre* has been collected nearly exclusively along the northern border of the Mediterranean basin (incl. adjacent islands), both in coastal habitats and inland (montane) habitats (see Figure 1). Its current distribution range includes: France (depts Hérault, Var, Bouches-du-Rhône, Gard), Italy (Sardinia, Gorizia, Livorno, Syracuse, Taranto, Veneto), Slovenia (see Scopoli 1763), Montenegro (Central Region), Croatia (Dubrovnik-Neretva Co.), Greece (Ionian and North Aegean Islands, Serres, Thessaloniki, Trikala), Bulgaria (Burgas), and Algeria (Oran). Its range overlaps with that of *O.sabulosum* only in Oran (Algeria), and on the islands of Sardinia (Italy) and Lesvos (Greece). In the latter site, both species have been collected (in different years by different collectors) in the same area.

Previous records from Austria, Ireland, Madeira or Crimea (Ukraine) could not be verified due to a lack of specimens, but it is very likely that the Irish and Madeiran records refer to *O.sabulosum* (see Figure 1).

##### Ecology.

With only two clear exceptions, *O.lacustre* has been recorded from mostly lowland locations in a 25 km zone along the Mediterranean coast, where it seems to occur along inland lakes which also corresponds with the description of the habitat of the type specimens (Scopoli 1763). Only on Sardinia (at 480m) and in Greece (prov. Serres) has the species been collected in habitats less or not affected by the sea. At the two Greek sites, both above 1,100m, and presumably also in Sardinia, the species occurred along small streams in mixed forest (beech and spruce forest in Greece).

#### Orthoceratium sabulosum

Taxon classificationAnimaliaDipteraDolichopodidae

(Becker, 1907)

Figs 1 , 3C–D , 4C, E, G , 5C , 6C , 8 , 9 , 10A

Alloeoneurus sabulosus Becker, 1907: 112. Type locality: Tunis (Tunisia) [ZMHB] – Alloeoneurus presumably synonymized with Orthoceratium by Kertész (1909)
Musca formosa Haliday, 1832: 356. Type locality: Cheshire (Great Britain) [unknown] **syn. n.**
Medeterus viridipes Macquart, 1834: 452. Type locality: Bordeaux (France) [unknown] **syn. n.**

##### Notes on synonyms.

*Muscaformosa* Haliday, 1832 and *Medeterusviridipes* Macquart, 1834 were previously listed as synonyms of *O.lacustre* by Becker (1917). Our attempts to retrieve and examine the specimens proved unsuccessful (the latter specimen could not be located, Christoph Daugeron, pers. comm.). However, taking the position of their capture localities into account (see Figure 1), there is little doubt that they are conspecific to *O.sabulosum*.

**Diagnosis.** Rather large, short-bodied, rather slender (but stouter than *O.lacustre*), brilliant green species with abdomen 1.3 × as long as thorax (Fig. 3C–D). All legs mainly dark and metallic with narrowly yellow knees. Wing smokey reddish yellow, with reddish yellow veins (Figure 5C). Apical section of vein M_1_ with strong sinuous bend at ½. Posterior border of wing indented at vein CuA_1_. Coxa I with strong white pubescence, and with one strong curved black bristle at basal 1/3 and three black bristles at apex (Figs 3C–D). Coxa II with one to three small black apical bristles on anterior face. Pedicel with strong apical bristles, with some ventral ones 1.5 × as long as pedicel is deep (Figure 5G). Ac uniseriate, rather strong, some about ½ × as long as dc. Male: face at least 1.4 × as wide as postpedicel is long (Figure 4C). Postpedicel with variable shape, at most as long as deep (Figure 4E). T_V_ with tapering ventral process at each side baring coalescent bristles (Figure 10A). Femur I with large avoid brownish spot in basal 2/5, about 1/4 of femur length, covered with a conspicuous yellow pubescence (Figure 6C). Femora I–II bare ventrally. Tibia II with two ad bristles, rarely with 3^rd^ much shorter basal bristle, and with two av bristles. Tibia III with two strong and two small pd bristles. Tarsus I with only one claw, and tarsomere taI_1_ with metallic reflection.

##### Redescription.

**Figure 8.**
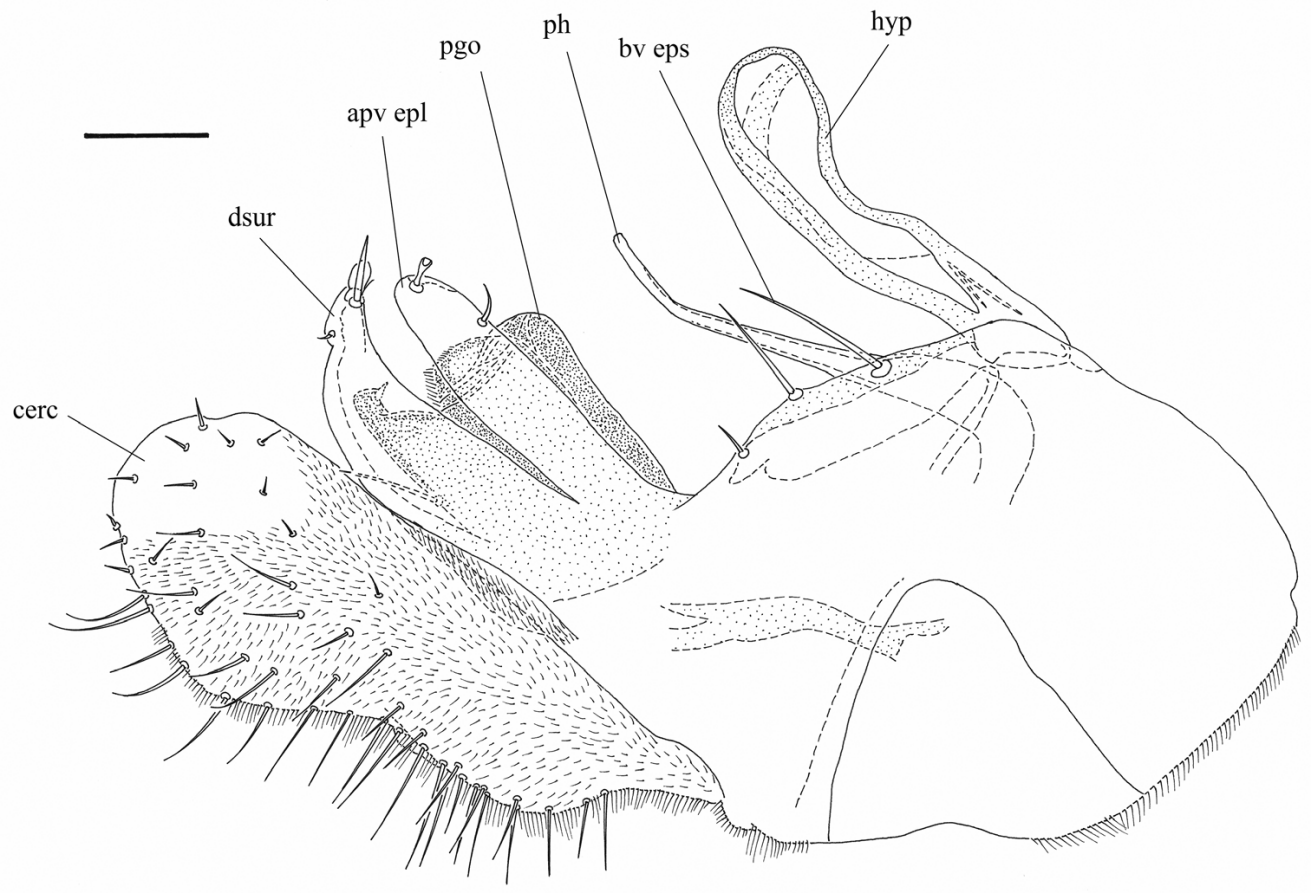
*Orthoceratiumsabulosum*, hypopygium (left lateral view). Abbreviations: hyp: hypandrium, ph: phallus, bv eps: basoventral epandrial setae, apv epl: apicoventral epandrial lobe, dsur: dorsal surstylar lobe, pgo: postgonites, cerc: cercus. Scale bar: 0.1 mm.

**Figure 9.**
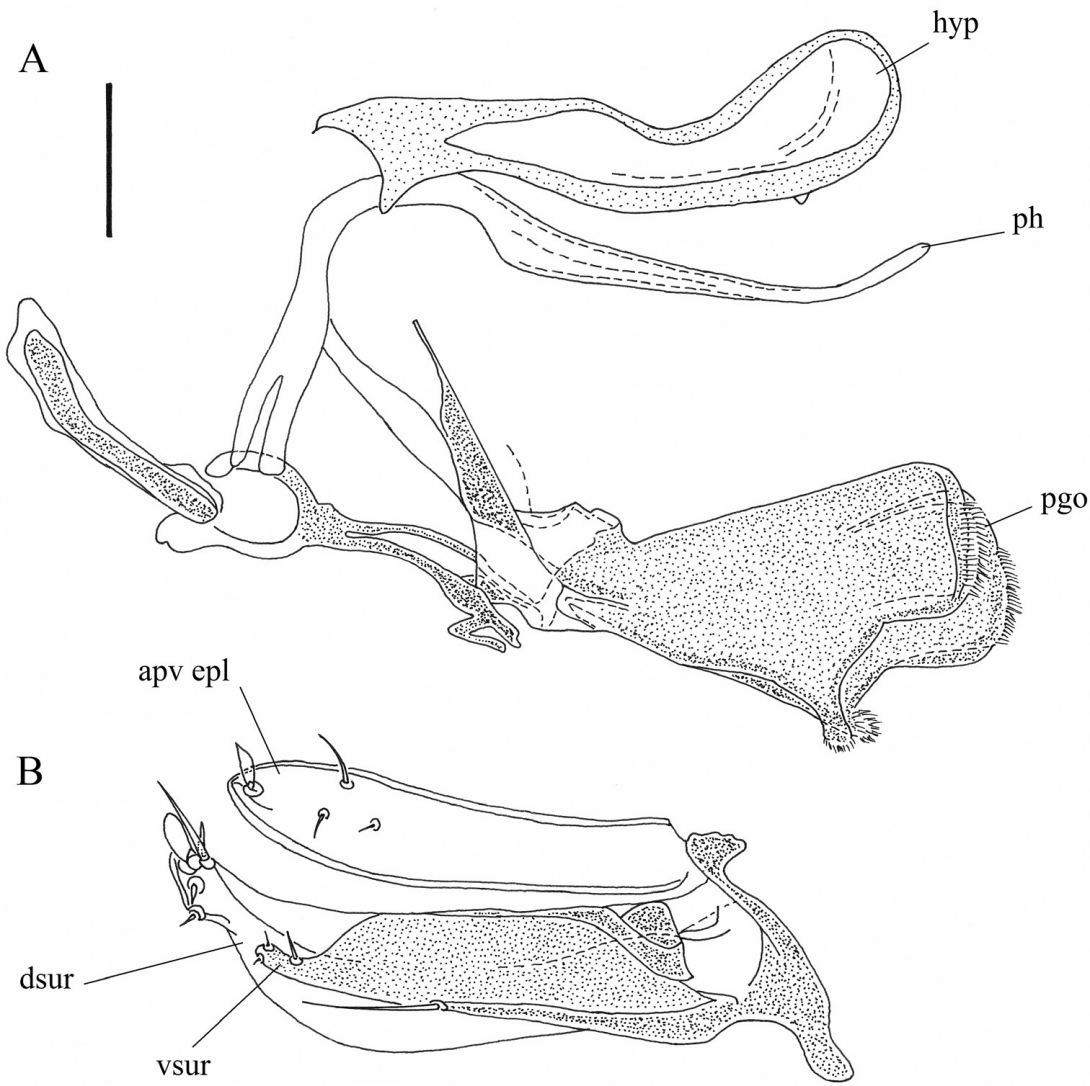
*Orthoceratiumsabulosum*, hypopygial appendages: **A** hypandrium (hyp), phallus (ph) and postgonites (pgo) (right lateral view) **B** apicoventral epandrial lobe (apv epl), dorsal (dsur) and ventral surstylar lobes (vsur) (inner view of left surstylus). Scale bar 0.1 mm (applicable for **A, B**).

**Figure 10.**
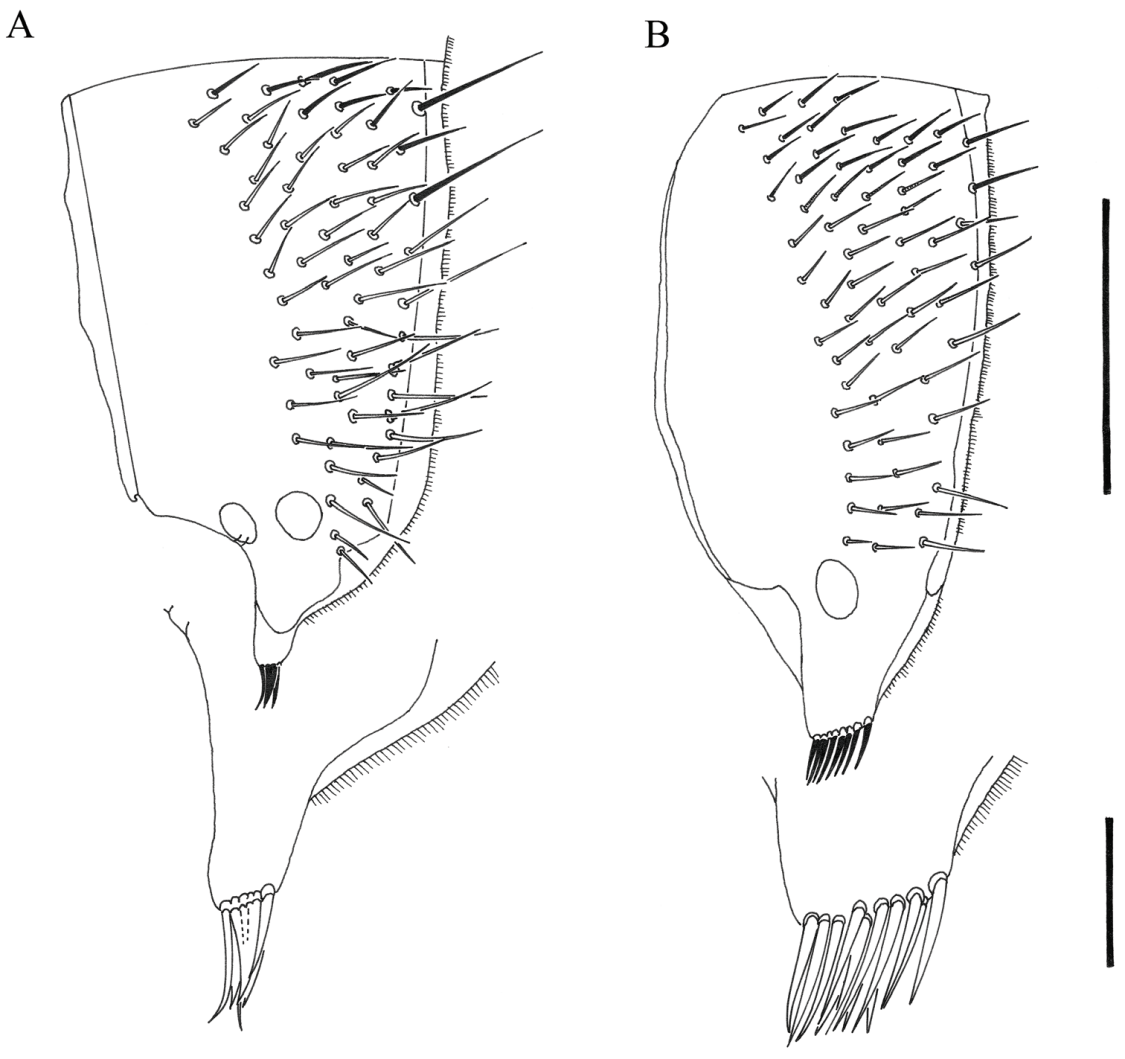
Posteroventral process on 5^th^ tergite, male: **A***O.sabulosum***B***Orthoceratiumlacustre*. Scale bars: 0.5 mm (tergites) and 0.1 mm (processes).

**Male**. Body length: 5.5–6.2 mm (n = 54); wing length: 5.2–6.1 mm (n = 77), 0.3 × as wide as long (n = 15) mm. **Head** (Fig. 4C, E, G). Face silvery white, rather parallel-sided, clypeus with triangular lower margin, strongly projecting; face 1.4–1.8 × as wide as postpedicel (length), with short white pubescence. Frons with metallic green ground colour, dusted yellowish white, less on posterior 1/2 in some specimens. Occiput brilliant metallic green, with weak dusting in some specimens, convex in middle. Palp about 1/4–1/3 of eye, trapezoid – elongate triangular, dark brown, dusted whitish, with white pubescence, and apical bristle absent. Proboscis dark brown. Eyes red, with short white pubescence. Uppermost eight-eleven postocular bristles bristles erect, black, and lower bristles curved, white, forming whiskers. One pair of black postocellar bristles. Antenna entirely dark brown, with scape bare, and pedicel with apical crown of strong bristles, with some ventral bristles about 1.5 × as long as pedicel is deep; postpedicel of rather variable shape, mostly rounded triangular, rarely rather subcircular, sometimes *Hydrophorus*-shaped, as long as deep, 0.7–0.8 × as long as scape and pedicel combined, with distinct pubescence; arista-like stylus dorsal, inserted at middle of upper rim of postpedicel, 2.3–2.7 × as long as first three antennal segments combined, bare. **Thorax** (Figure 3C). Mesonotum entirely brilliant metallic green with sometimes bluish violet tinge, strongly dusted greyish white on pleura and certain zones on dorsum, only without dusting between dc and ac, and between dc and npl areas; scutellum dark green to bluish green with violet tinge, bare on dorsum, with four marginal bristles, lateral pair much smaller than median pair. Anterior spiracle with group of multiple curved, yellowish white, long setae. Thoracic bristles black. Seven dc, with 1^st^dc laterally off-set, and 6–7^th^dc stronger; six to nine ac, uniseriate, reaching till 5^th^dc, rather strong, some about 1/2 × as long as dc; with two strong black, and one minute white ant pprn, one internal and one external bas pprn, one psut ial, one sut ial, two npl, two spal, and one pal bristles. Upper proepisternum with a large group of long yellowish white curved setae; lower proepisternum with one strong black curved bristle and a small group of yellowish white curved setae. **Wing** (Figure 5C). Slightly smokey reddish yellow, with reddish yellow veins. Vein R_4+5_ sinuous near wing apex, there parallel with vein M_1_; apical section of vein M_1_ with strong sinuous bend at ½ (MSSC); crossvein dm-cu slightly concave; posterior border of wing indented at vein CuA_1_. Proximal section of vein M_1_ 2.0 × as long as apical section. Proximal section of vein CuA_1_ 7.1 × as long as apical section. CuA_x_ ratio: 1.6. Halter pale, calypteral fringe yellowish white. **Legs** (Figs 3C, 6C). Overall dark, metallic green, with pale yellow knees in all legs, and with black bristles. Coxae I–III dark, with metallic green ground colour, strongly dusted whitish, coxae I and III with extreme apex, and coxa II with less than apical 1/4 yellow. Coxa I with dense, white pubescence and one strong black curved bristle at basal 1/3, about 0.5 × as long as coxa I is long, and three strong, black ap bristles. Coxa II with dense white pubescence on anterior face, one inclined black bristle at 1/2, and one to three smaller black bristles at apex; lateral face bare. Coxa III with black, erect external bristle, inserted at 1/2, with vertical row of white setae. Trochanters dark brown. Femora I–III brilliant metallic green, femora I–II with pale yellow knee on apical 1/8, and on apical 1/10 in femur III. Femur I with large avoid brownish pv spot in basal 2/5, about 1/4 of femur length, covered with a conspicuous yellow pubescence (MSSC); with one rather small pv preapical bristle. Femur II with one strong ad bristle, at less than apical 1/5, and one small pv preapical bristle; with one row of short inclined pv setae along entire length, white on basal 2/3 and black on apical 1/3, longest at basis and apex. Femur III with one strong ad bristle, at about apical 1/3, and with one small pv preapical bristle; with one thin erect black ds bristle at about basal 1/5, nearly 0.5 × as long as femur is deep. Tibia I brilliant metallic green, and tibia I with basal 1/8, tibia II with basal 1/9, and tibia III with basal 1/10 pale yellow. Tibia I with two ds bristles, 2–3 × as long as tibia is deep; with two small ad bristles, 1–1.5 × as long as tibia is deep, and with three pv bristles, 2 × as long as tibia is deep; with white pilosity on av face along entire length, and with two small ap bristles. Tibia II with two ad bristles, 4 × as long as tibia is deep, rarely with 3^rd^ much shorter basal bristle; with two pd bristles, about 2.5 × as long as tibia is deep, with basal bristle shorter, and with four ap bristles; with two av bristles, at basal 1/3 and 2/3, 2 × and 1.5 × as long as tibia is deep resp.; with one pv bristle, at basal 1/6, 2 × as long as tibia is deep, and two small pv bristles in apical 1/2, not as long as tibia is deep. Tibia III with five ad bristles, about 3 × as long as tibia is deep, two strong and two small pd bristles, former about 3.5 ×, latter 2 × as long as tibia is deep, and four strong ap bristles; with distinct pd row on apical 1/2; with four to five av bristles, 1.5 × as long as tibia is deep, and multiple shorter pv setae along entire length. Tarsi I–III black, with taI–III_1_ with metallic green to bluish reflection and with multiple short black vt bristles, nearly as long as taI–III_1_ is deep; taI_5_ with long curved dorsal setae at apex, 0.8 × as long as taI_5_ is long; only inner claw present (MSSC). Ratio of femur/tibia/tarsomeres 1–5 in leg I: 9/10/5.4/2.1/1.5/1/1.1, in leg II: 9/11/8/3.5/2.3/1.2/1, and in leg III: 9.8/13.8/6/4.2/2.6/1.3/1. **Abdomen** (Figure 3C). Basal five segments pubescent, 6^th^ bare, minute, only visible dorsally; T brilliant metallic green, with bluish or with bronze tinge in some specimens, strongly dusted whitish on lower margins, with short dense black pubescence on dorsum, with long yellowish white setae laterally, strongest on sides of T_I_, and T_I–V_ with black bristles on posterior margin, strongest on T_I_; T_V_ brilliant metallic green with prominent dark bristles at posterior margin, with tapering ventral process at each side baring coalescent bristles (MSSC) (Figure 10A). ST with green ground colour and strong whitish dusting, with yellowish white, erect bristles. Hypopygium (Figures 8–9) with epandrium concolorous with tergites; hypandrium rather stout with subcircular apex, with ventrally curled up sides forming a gutter; phallus slender and strongly curved; two larger and one smaller basoventral epandrial setae, apicoventral epandrial lobe pale to reddish brown, stout, elongate ovoid and close to outer surstylar lobe; surstylus with robust pale to reddish brown outer (or dorsal) lobe with subcircular bristle at apex, inner (or ventral) surstylar lobe dark, robust with tapering apex baring a few bristles; postgonites robust, dark, with apical pubescence and a ds process; cercus dark brown, medium-sized, rather rectangular, with apex nearly bare. **Female**. Body length: 6.4–6.6 mm (n = 47); wing length: 5.2–6.5 mm (n = 65), 0.3 × as wide as long (n = 15). As male, except for: abdomen 1.4 × as long as thorax. Face 2.0–2.3 × (n = 5) as wide as postpedicel (length). Palp about 1/5–1/4 of eye, ovoid. Uppermost six to nine postocular bristles erect, black. One pair of postocellar bristles, rarely with two pairs. Pedicel with some ventral bristles longer than pedicel is deep; postpedicel 0.8–1.0 × as long as deep; arista-like stylus 2.4–2.7 × as long as first three antennal segments combined. Thorax with five to eight ac, reaching between 4^th^ and 5^th^dc. Vein R_4+5_ bended but straight near wing apex, there parallel with vein M_1_; apical section of vein M_1_ with weak bend (sinuous) at 1/2; crossvein dm-cu rather straight. Proximal section of M 1.7 × as long as apical section. Proximal section of CuA_1_ 6.5 × as long as apical section. CuA_x_ ratio: 1.5. Femur I bare ventrally. Femur III with two to four thin erect black bristles in basal 1/5, about 1/3 × as long as femur is deep. Tibia II with two large ad bristles, and often 3^rd^ shorter basal bristle. Tarsus III black, unmetallic, taI_1_ with multiple short black ventral bristles ventrally, nearly as long as taI_1_ is deep; taI_5_ with both claws. Ratio of femur/tibia/tarsomeres 1–5 in leg I: 8.5/9/5.3/2/1.5/1/1, in leg II: 9/10.5/7.8/3.2/1.9/1/1, and in leg III: 10.1/14.5/6.2/4.2/2.4/1.2/1. Abdomen with five pubescent segments, 6^th^ invisible; also ST_IV_ with strong whitish dusting.

##### Type specimens examined.

**LECTOTYPE** (here designated to fix the identity of the species) ♂, **TUNISIA**: [Tunis governate] “Tunis904” / “Ujhelyi”, [bottom side] “X 26”; “*Alloeoneurus*” / “*sabulosus* Beck.” / “det. Becker”; [red rectangular] “Lectotypus”; “Zool. Mus.” / “Berlin” [ZMHB] (TN-01). **PARALECTOTYPE** ♀, **TUNISIA**: “Tunis904” / “Ujhelyi”, [bottom side] “XI 2”; [red rectangular] “Typus”; “Zool. Mus.” / “Berlin” [ZMHB] (TN-01).

##### Notes on type material.

The original description of this species by Becker (1907) is based on multiple specimens (males and females) from Tunis, present in the HMNH at the time of the description. A holotype was not formally designated in Becker (1907) and it remains uncertain if the two specimens from the ZMHB were part of the type series. Fact is that both specimens in the ZMHB were collected by Ujhelyi in 1904 in Tunis, where Biró (HMNH) also collected Diptera in 1903 (Horn et al. 1990). And as indicated by Becker (1906) all Tunis specimens were examined at the same time which led to the description of the species in 1907. It is thus very likely that the ZMHB specimens, indeed, belonged to the type series. As it remains uncertain who attached the existing lectotype and paralectotype lables to the ZMHB specimens and when, a formal designation is provided here. We have no explanation, though, how they ended up in Becker’s collection (ZMHB). The Becker catalogues at the ZMHB list a few specimens that he received as a gift from Biró, but these specimens always carry according labels (Jenny Pohl, pers. comm.). However, no such labels were found on the current lectotype and paralectotype.

##### Other material examined.

See Suppl. material 1. List of (non-type) records of *Orthoceratium*.

##### Distribution.

**Figure 11.**
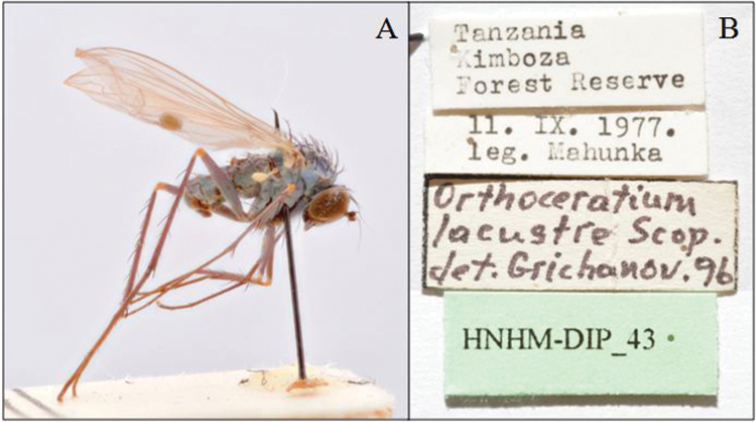
*Orthoceratiumsabulosum* from Kimboza Forest Reserve, Tanzania: **A** male specimen **B** labels (photos by Zoltán Soltész).

Compared to *O.lacustre*, *O.sabulosum* is much more widespread in the West Palaearctic and currently known with certainty from 14 countries, although it has not (yet) been collected in the northern part of the Mediterranean basin (see Figure 1): Denmark (South Jutland), Germany (Niedersachsen), Netherlands (Friesland, Zeeland, Zuid-Holland), Belgium (West-Vlaanderen), Great Britain (Cornwall, Dorset, Essex, Glamorganshire, Kent, Norfolk, North Somerset, Suffolk, Cheshire?), Ireland?, France (Morbihan, Gironde?), Portugal (Algarve, Beira Alta, Beira Litoral, Douro Litoral), Madeira?, Spain (Alicante, Cádiz, Córdoba, Segovia, Teruel, Zaragoza-Soria), Italy (Sardinia), Greece (Attica, North Aegean Islands), Algeria (Algiers, El Tarf, Oran), Tunisia (Ben Arous, Jendouba, Tunis), Turkey (inner Western Anatolia) and Iran (East Azerbaijan). Previous doubts about the occurrence of *Orthoceratium* in subsaharan Africa proved incorrect. However, specimens of *O.lacustre* identified and recorded by Grichanov (1997) from Tanzania, in fact, proved to belong to *O.sabulosum* (Figure 11). These records (see Appendix) from inland forest areas far beyond its West Palaearctic distribution range remain unexplained.

##### Ecology.

Based on the current records, the distribution range of *O.sabulosum* differs significantly from that of *O.lacustre*. Despite that, however, both species seem to display a surprisingly similar ecological amplitude. In northwestern Europe (from Great Britain over Belgium and the Netherlands to Germany and Denmark), it is confined to humid coastal habitats, with a strong preference for salt marshes and brackish marshes. In Belgium, the species has only been collected in sea-aster (*Astertripolium*) vegetations, bordering shallow brackish to saltwater ponds (Pollet et al. 2017) and in wet to slightly flooded *Salicornia* vegetations in brackish marshes and salt marshes (Pollet, unpubl. data). Also in Greece (Lesvos), Portugal (Algarve, Douro Litoral), Spain (Alicante) and north Africa (Algeria, Tunisia), records originate from locations close to the sea. In sharp contrast, the species is also known from locations between 700 m and 1,907 m in Portugal, Spain, Turkey and Iran, mostly in (coniferous) forest habitat and often with or close to small streams or open water (lakes).

### Key to species of *Orthoceratium* Schrank (both sexes)

**Table:** 

1	Coxa I with one strong curved black bristle at basal 1/3 (Figure 3C). Coxa II with one to three small black apical bristles on anterior face. Pedicel with strong apical bristles, with some ventral ones 1.5 × as long as pedicel is deep (Figure 4G). Ac rather strong, some about ½ × as long as dc. Male: face at least 1.4 × as wide as postpedicel is long (Figure 4C). Postpedicel mostly rounded triangular, at most as long as deep (Figure 4E). T_V_ with prominent dark bristles at posterior margin, and tapering ventral process at each side baring coalescent bristles (Figure 10A). Femur I with large avoid brownish pv spot in basal 2/5, about 1/4 of femur length, covered with a conspicuous yellow pilosity (Figure 6C). Femora I–II bare ventrally. Tibia II with two ad bristles, rarely with 3^rd^ much shorter basal bristle, and with two av bristles. Tibia III with two strong and two small pd bristles. Tarsomere taI_1_ with metallic reflection	***O.sabulosum* (Becker, 1907)**
–	Coxa I without a black bristle at basal 1/3 (Figures 2, 3A). Coxa II with only pale bristles at apex anteriorly. Pedicel with short apical bristles (Figure 4F). Ac rather small, at most 1/3 × as long as dc. Male: face not as wide as postpedicel is long (Figure 4A). Postpedicel elongate triangular, at least 1.2 × as long as deep (Figure 4D). T_V_ without prominent dark bristles on posterior margin, with blunt ventral process at each side with short dark pubescence (Figure 10B). Femur I with small ovoid brownish yellow pv tuft just beyond basal 1/4, about 1/8 of femur length (Figure 6A). Femora I–II with multiple rows of very short white erect setae on basal ½. Tibia II with three ad bristles, with basal bristle shorter, and with one av bristle. Tibia III with four strong and one small pd bristles. Tarsomere taI_1_ mostly unmetallic	***O.lacustre* (Scopoli, 1763)**

### Biometrics

Table 1 gives a summary of the wing lengths measured in 76 and 142 specimens of *O.lacustre* and *O.sabulosum*, resp. On average, wings in males (+ 0.1 mm) and females (+ 0.2 mm) of *O.lacustre* are only very slightly longer than in *O.sabulosum*. Indifferently, both overall and in separate populations or datasets per country, wings in males of both species were approximately 0.5 mm shorter than in females. Size variations within the same sex in separate populations mostly proved higher in the females, with a maximum of 0.9 mm in males of Greece and even of 1.5 mm in females of France, both in *O.lacustre*. Size differences between both species in the keys by Becker (1907) and Negrobov (1979) hereby prove unreliable.

## Discussion

Considering the fair size of *Orthoceratium* – compared to other dolichopodid lineages, it remains surprising that key features of this genus have been overlooked by previous authors. The ventral process of the 5^th^ tergite was only mentioned by Becker (1907) and Negrobov (1979) – incorrectly as 4^th^ sternite! – and the posteroventral spot on the fore femur only by Parent (1938). In describing *O.sabulosum*Becker (1907) even omitted the most decisive character to separate this species from *O.lacustre*, i.e. the strong black bristle on the fore coxa. All authors ignored the presence of one single claw of the fore tarsus in the male and Negrobov (1979) even emphasized that claws are well developed.

Even more worrisome is the fact that Becker (1907) seems to have mixed up both species in the original description of *O.sabulosum*. The description of the ventral process of the 5^th^ sternite “… aber der vierte Bauchring ist anders und hier [in *O.sabulosum*] ganz einfach gebildet, während er bei *All.lacustris* spitz dreieckig endigt und vorsteht …” clearly points towards *O.lacustre*. This erroneous interpretation was copied by Negrobov (1979). The latter assumption seems to be confirmed by the description of the hypopygium and the cercus by Becker (1907): “… an den Seiten des Hinterleibes, am Bauche und auf dem grau bestäubten Hypopygium stehen zarte weisse Haare.” Indeed, the cercus is only clearly pubescent in *O.lacustre*.

Unlike Negrobov (1978, 1979), Parent (1938) clearly used specimens of *O.sabulosum* in his description of *O.lacustre*, which must have been simple misfortune. In fact, Parent listed records of his ‘*O.lacustre*’ from localities in the north of France (Pas-de-Calais, Morbihan) and the south (Hyères, Hérault), two regions where either *O.sabulosum* or *O.lacustre* have been recorded, resp. (see Figure 1). Apparently, he had no idea that he was dealing with two different species.

But most of all, it is remarkable that *O.sabulosum* has been ignored as a species since its description. Indeed, apart from its Tunis type locality and its inclusion in the Becker (1917) and Negrobov (1979) keys, this species has further never been mentioned in the literature. As a result, all Diptera workers dealing with Palaearctic dolichopodids automatically considered any *Orthoceratium* they encountered being *O.lacustre*. That group of dolichopodid workers even includes esteemed contemporary colleagues or peers from this and the previous century: CE Dyte, H Ulrich, O Parent, and H Meuffels. Also the senior author misidentified *O.sabulosum* as *O.lacustre* when he termed the species “Extinct in Flanders” (Pollet 2000) and when he recently reported on its rediscovery (Pollet et al. 2017).

One wonders how this series of mistakes could happen and last for over 250 years? A first reason seems to be that no previous researcher made the effort to compare with the type specimens, not even in the post-World War II era. Next to the fact that the type of *O.lacustre* was lost, most dolichopodid workers seemed to believe that *O.sabulosum* was a strictly southern Mediterranean species. With the present study we clearly proved that this species has a considerably larger distribution range. A second equally important reason are the insignificant, even misleading, characters used in species descriptions and identification keys, and the mere copying of those keys by subsequent authors. Scopoli (1763) described the eyes as green, while they are clearly red (see Figs 2, 4A, B, C) and stressed – for unknown reasons – the colour of the tarsal pulvilli. Of the five characters used in the keys by Becker (1917) and Negrobov (1979), only the face width proves to be diagnostically significant. In contrast, as can be concluded from the key given in the present paper, in males as well as in females a number of reliable features can be found to separate both species. But, of course, this requires a detailed examination and characterization of the species.

Some authors tend to build elaborate and detailed species descriptions (see e.g., Brooks and Ulrich 2012, Pollet et al. 2015, Kazerani et al. 2017) using a large number of characters (173 in the two latter papers) in order to avoid misidentifications (or to correct misinterpreted species identities from the past). This type of tedious time-consuming work is sometimes considered unnecessary or unwanted by peers for the simple reason that it might prevent researchers tackling the current and ever growing taxonomic impediment (Wheeler et al. 2004; Borkent et al. 2017), and this statement certainly contains some truth. However, it should not be forgotten how much (precious) time is invested not only to correct taxonomic mistakes from the past (e.g., current paper, Kazerani et al. 2017), but also to verify the identity of species on the basis of insufficiently detailed descriptions without type material at hand. In our opinion, a description should stand on itself and must allow its user to decide unequivocally whether he is dealing with the described species or not. In case the species description is not satisfactory, and at present, this is very often the case, the only solution is the examination of the type material.

Here lies the importance of type material that cannot be underestimated. Only after the examination of the types of *O.sabulosum* did we realize that researchers had been misidentifying *O.lacustre* for more than 2.5 centuries (and every correct identification might merely be considered as a fortunate coincidence). Indeed, only when the type locality of *O.lacustre* proved to be situated within the distribution range of ‘species B’, it became apparent that both species were conspecific. If this would not have been the case, and assuming that the face width would have been unreliable like the other four features used in the extant identification keys, *O.lacustre* might as well have rendered *nomen dubium*, and ‘species B’ might have been described as new. In this respect, the use of alternatives to physical type specimens deposited in a museum (and photos thereof) like e.g., field images of uncollected specimens as promoted by Minteer et al. (2014) and applied by Marshall and Evenhuis (2015) would most likely not have solved this issue. Therefore, we strongly support the strict interpretation of Article 16.4.2 of the ICZN Code (ICZN 1999).

## Conclusion

For more than 2.5 centuries the *Orthoceratium* species that occurs in northwestern Europe has indifferently been considered as *O.lacustre*, while it was, in reality, *O.sabulosum*. This is all the more surprising since *Orthoceratium* species are among the larger and more conspicuous dolichopodid species in Europe. The main reasons for this continuous series of misinterpretations seemed to be the mere copying of keys by successive authors that contained misleading information, the omission of examining type specimens of *O.sabulosum*, and the loss of the type specimens of *O.lacustre*. The importance of type specimens and the examination thereof is stressed, as well as that of unequivocal, detailed, and well-illustrated descriptions to avoid this kind of taxonomic confusion.

## Supplementary Material

XML Treatment for Orthoceratium lacustre

XML Treatment for Orthoceratium sabulosum

## Supplementary materials

Supplementary material 1 List of (non-type) records of *Orthoceratium* Data type: species data This is an open access article distributed under the terms of the Creative Commons Attribution License (CC BY 4.0), which permits unrestricted use, distribution, and reproduction in any medium, provided the original author and source are credited.Author: Marc Pollet
