# Supplementary material for: The quest for the identity of Orthoceratiumlacustre (Scopoli, 1763) reveals centuries of misidentifications (Diptera, Dolichopodidae)
Source: Zookeys. 2018 Aug 16;(782):49–79. doi: 10.3897/zookeys.782.26329 (PMC6160834; doi:10.3897/zookeys.782.26329)
Supplement: Supplementary material 1 — List of (non-type) records of Orthoceratium [file zookeys-782-049-s001.pdf]

Supplementary material 1. List of (non-type) records of *Orthoceratium*

The quest for the identity of *Orthoceratium lacustre* (Scopoli, 1763) reveals centuries of misidentifications (Diptera, Dolichopodidae)

Marc Pollet & Andreas Stark

Citation: *Pollet M, Stark A (2018) The quest for the identity of Orthoceratium lacustre (Scopoli, 1763) reveals centuries of misidentifications (Diptera, Dolichopodidae). ZooKeys 782: 49–79. https://doi.org/10.3897/zookeys.782.26329*

Orthoceratium lacustre (Scopoli, 1763)

(BG-01) BULGARIA: 1♂, 1♀, Burgas, Burgas, Atanasovsko Ezero, 11.v.1967 - labels: "Burgas. At. es. ♂" / "11.5.1967, *Skalicorn*" / "V. Beschovsky ♀"; [label in Cyrillic]; "*ORTHOCERATIUM* ♂" / "*lacustre* (SCOPOLI, 1763)" / "Beschovski det. ♀"; "2."; 1♂, 1♀, same data - labels: "Burgas. At. es. ♂" / "11.5.1967, *iuncvs*" / "V. Beschovsky ♀"; [label in Cyrillic]; "*ORTHOCERATIUM* ♂" / "*lacustre* (SCOPOLI, 1763)" / "Beschovski det. ♀"; "12."; 3♂, same data - labels: "Burgas. At. es. ♂♂" / "11.5.1967, *Skalicorn*" / "V. Beschovsky"; [label in Cyrillic]; "*ORTHOCERATIUM* ♂♂" / "*lacustre* (SCOPOLI, 1763)" / "Beschovski det."; "8."; 2♀, same location and collector, 17.v.1967 - labels: "Burgas. At. es." / "17.5.1967, *Salcornia*" / "V. Beschovsky ♀"; [label in Cyrillic]; "*ORTHOCERATIUM*" / "*lacustre* (SCOPOLI, 1763)" / "Beschovski det. ♀♀"; "16."; 2♀, same data - labels: "Burgas. At. es. ♀" / "17.5.1967, *Salcornia*" / "V. Beschovsky"; [label in Cyrillic]; "*ORTHOCERATIUM* ♀" / "*lacustre* (SCOPOLI, 1763)" / "Beschovski det. ♀"; "11."; 2♂, same data - labels: "Burgas. At. es. ♂" / "17.5.1967, *Salcornia*" / "V. Beschovsky"; [label in Cyrillic]; "*ORTHOCERATIUM* ♂" / "*lacustre* (SCOPOLI, 1763)" / "Beschovski det."; "4."; 1♂, 1♀, same data - labels: "Burgas. At. es. ♂" / "17.5.1967, *Salcornia*" / "V. Beschovsky ♀"; [label in Cyrillic]; "*ORTHOCERATIUM*" / "*lacustre* (SCOPOLI, 1763)" / "Beschovski det. ♂♀"; "1."; 2♂, same data - labels: "Burgas. At. es. ♂♂" / "17.5.1967, Salcornia" / "V. Beschovsky"; [label in Cyrillic]; "*ORTHOCERATIUM* ♂♂" / "*lacustre* (SCOPOLI, 1763)" / "Beschovski det."; "5."; 1♀, same data - labels: "Burgas. At. es. ♀" / "17.5.1967, Salcornia" / "V. Besahovsky ♀"; [label in Cyrillic]; "*ORTHOCERATIUM* ♀" / "*lacustre* (SCOPOLI, 1763)" / "Beschovski det."; "13."; 1♀, same data - labels: "Burgas. At. es. ♀" / "17.5.1967, *iuncvs*" / "V. Beschovsky"; [label in Cyrillic]; "*ORTHOCERATIUM*" / "*lacustre* (SCOPOLI, 1763)" / "Beschovski det. ♀"; "10."; 1♂, same data - labels: "Burgas. At. es. ♂" / "17.5.1967, Salcornia" / "V. Besahovsky"; [label in Cyrillic]; "*ORTHOCERATIUM* ♂" / "*lacustre* (SCOPOLI, 1763)" / "Beschovski det."; "9."; [remarks:]; 1♀, same location and collector, 30.x.1967 - labels: "Burgas. At. es. ♀" / "30.10.1967, *Salcornia*" / "V. Besahovsky"; [label in Cyrillic]; "*ORTHOCERATIUM*" / "*lacustre* (SCOPOLI, 1763)" / "Beschovski det. ♀"; "14.", all leg. V. Beschovski [IBER (collection Beschovski)] [note: *Salcornia* and *Skalicorn* refer to *Salicornia*, and *iucvs* to *Juncus*];

(BG-02) BULGARIA: 2♀, Burgas, Burgas, 17.v.1967 - labels: "Burgas" / "17.5.1967, iuncus" / "V. Beschovsky ♀"; [label in Cyrillic]; "*ORTHOCERATIUM*" / "*lacustre* (SCOPOLI, 1763)" / "Beschovski det. ♀♀"; "15."; 2♂, same data - labels: "Burgas ♂" / "17.5.1967, iuncus" / "V. Beschovsky"; [label in Cyrillic]; "*ORTHOCERATIUM*" / "*lacustre* (SCOPOLI, 1763)" / "Beschovski det. ♂♂"; "7."; 2♂, same

data - labels: "Burgas ♂♂" / "17.5.1967, iuncus" / "V. Beschovsky"; [label in Cyrillic]; "*ORTHOCERATIUM* ♂♂" / "*lacustre* (SCOPOLI, 1763)" / "Beschovski det."; "6."; 1♂, same data - labels: "Burgas" / "17.5.1968, Salicorn" / "V. Beschovsky ♂"; [label in Cyrillic]; "*ORTHOCERATIUM* ♂" / "*lacustre* (SCOPOLI, 1763)" / "Beschovski det."; "3.", all leg. V. Beschovski [IBER (collection Beschovski)] [note: *iucus* refers to *Juncus*];

(DZ-04) - ALGERIA: 1♀, Oran [NHMW] - labels: "Schmiedeknecht" / "Oran 1895";

(HR-01) - CROATIA: 1♀, Dubrovnik-Neretva County, Ston, leg. T. Becker [ZMHB (collection Becker)] - labels: [brownish grey rectangular] "Dalmatien" / "18/5 22578"; "Zool. Mus." / "Berlin" [remarks: Becker catalogue: 22578: "*Alloconeurus lacustris* Scopoli, Stagno [= Ston], Salinen am Meer, Dalmatien", deposited: 18. Mai 1889];

(FR-01) - FRANCE: 1♂, Bouches-du-Rhône (dept.), Stes Maries-de-la-Mer, Camargue, 9.v.1946, leg. A. Bayard [MNHN (collection générale)] - labels: "Stes MARIES-de-la-MER" / "Camargue 9 mai 1946" / "Coll. A. Bayard"; "*Sciopus* ♂" / "??"; "*Orthoceratium* ♂" / "*lacustre* (Scopoli)" / "H. Ulrich det. 1983";

(FR-03) - FRANCE: 1♂, Hérault (dept.), Montpellier, 1.vi.1925, leg. [MNHN (collection Parent)] - labels: "Montpellier" / "1.VI.25"; "O. lacustre" / "Scop";

(FR-04) - FRANCE: 10♂, 5♀, Var (dept.), Hyères, leg. T. Becker [ZMHB (collection Becker)] - labels: "Hyères J." / "48601"; "*lacustris*" / "Scop."; "Zool. Mus." / "Berlin" [remarks: Becker catalogue: 48601: "*Alloconeurus lacustris* Scopoli, Hyères, Salinen", deposited: 11. - 25. Mai 1902]; 1♂, same data - labels: "Hyères J." / "48601"; "*lacustris*" / "Scop"; "Zool. Mus." / "Berlin" [remarks: Becker catalogue: 48601: "*Alloconeurus lacustris* Scopoli, Hyères, Salinen", deposited: 11. - 25. Mai 1902]; 1♀, same data - labels: "Hyères J." / "48601"; "*lacustris*" / "Scop"; "Zool. Mus." / "Berlin" [remarks: Becker catalogue: 48601: "*Alloconeurus lacustris* Scopoli, Hyères, Salinen", deposited: 11. - 25. Mai 1902]; 1♂, Var (dept.), Hyères [NHMW] - labels: "Hyères" / "V" / "48601";

(FR-05) - FRANCE: 2♂, 2♀, Gard (dept.), Le-Grau-du-Roi, Etang de Ponara [= Ponant], 24.iv.2001, leg. A. Stark [ANSC] - labels: F, Gard, Le-Grau-du-Roi, Nordufer v. "Etang du Ponara [Ponant]", 24. April 2001, leg. et det. A. Stark (W);

(FR-06) - FRANCE: 1♂, Hérault (dept.), Pérols, [Parc du] Mas Rouge, 13.x.1966, leg. Harant [ZMFK] - labels: Perols / mas rouge / 13.X.1966; [upside down] *Orthoceratium* / *lacustre* ♂; [upside down] aus Coll. / HARANT; [upside down] *Orthoceratium* ♂ / *lacustre* (Scopoli) / H. Ulrich det. 1983; [upside down] ZFMK DIP / 00019812; 1♀, Hérault (dept.), Pérols, [Parc du] Mas Rouge, 13.x.1966, leg. Harant [ZMFK] - labels: Perols / Mas Rouge / 13/X.1966; [upside down] Orthoceratium / lacustre ♀; [upside down] aus Coll. / HARANT; [upside down] *Orthoceratium* ♀ / *lacustre* (Scopoli) / H. Ulrich det. 1983; [upside down] ZFMK DIP / 00019818; 1♀, Hérault (dept.), Pérols, [Parc du] Mas Rouge, 13.x.1966, leg. Harant [ZMFK] - labels: Perols / Mas rouge / 13.X.1966; [upside down] *Orthoceratium* / *lacustre*; [upside down] aus Coll. / HARANT; [upside down] *Orthoceratium* ♀ / *lacustre* (Scopoli) / H. Ulrich det. 1983; [upside down] ZFMK DIP / 00019819; 1♀, Hérault (dept.), Pérols, [Parc du] Mas Rouge, leg. Harant [ZMFK] - labels: Perols, / Mas rouge, / Sansouire; [upside down] aus Coll. /

HARANT; [upside down] *Orthoceratium* ♀ / *lacustre* (Scopoli) / H. Ulrich det. 1983; [upside down] ZFMK DIP / 00019523; 1♀, Hérault (dept.), Pérols, [Parc du] Mas Rouge, leg. Harant [ZMFK] - labels: Perols, / Mas rouge, / Sansouire; [upside down] aus Coll. / HARANT; [upside down] *Orthoceratium* ♀ / *lacustre* (Scopoli) / H. Ulrich det. 1983; [upside down] ZFMK DIP / 00019524; 1♀, Hérault (dept.), Pérols, [Parc du] Mas Rouge, leg. Harant [ZMFK] - labels: Perols, / Mas rouge, / Sansouire; [upside down] aus Coll. / HARANT; [upside down] *Orthoceratium* ♀ / *lacustre* (Scopoli) / H. Ulrich det. 1983; [upside down] ZFMK DIP / 00019525; 1♀, Hérault (dept.), Pérols, [Parc du] Mas Rouge, leg. Harant [ZMFK] - labels: Perols, / Mas rouge, / Sansouire; [upside down] aus Coll. / HARANT; [upside down] *Orthoceratium* ♀ / *lacustre* (Scopoli) / H. Ulrich det. 1983; [upside down] ZFMK DIP / 00019526; 1♀, Hérault (dept.), Pérols, [Parc du] Mas Rouge, leg. Harant [ZMFK] - labels: Perols, / Mas rouge, / Sansouire; [upside down] aus Coll. / HARANT; [upside down] *Orthoceratium* ♀ / *lacustre* (Scopoli) / H. Ulrich det. 1983; [upside down] ZFMK DIP / 00019527;

(**GR-02**) - **GREECE**: 1♂, North Aegean Islands, Lesvos, 4.8 km NE Achladeri ("Derbyshire"), 18.v.2000, SW, leg. Marc Pollet [MAPC] (W);

(**GR-03**) - **GREECE**: 1♂, 1♀, Thessaloniki, Mikra, iv.1918 [MNHN (collection générale)] - labels: "MUSEUM PARIS" / "Env. de SOLONIQUE" / "MIKRA" / "ECOLE d'AGRICULTURE" / "GRECQUE" / "Armée d'ORIENT 1918"; "AVRIL"; "*Orthoceratium*" / "*lacustre* Scop " / "det O Parent";

(**GR-04**) - **GREECE**: 1♂, Thessaloniki, Thessaloniki [ZMHB (collection Becker)] - labels: "Saloniki" / "26242"; "Zool. Mus." / "Berlin” [remarks: slightly damaged during transport; Becker catalogue: 26242: "*Alloconeurus lacustris* Scopoli, Saloniki, Türkei", deposited: 25. Mai 1890]; 1♂, Thessaloniki, Thessaloniki, leg. T. Becker [ZMHB (collection Becker)] - labels: "Saloniki" / "26242"; "Zool. Mus." / "Berlin" [remarks: Becker catalogue: 26242: "*Alloconeurus lacustris* Scopoli, Saloniki, Türkei", deposited: 25. Mai 1890]; 1♀, Thessaloniki, Thessaloniki, leg. T. Becker [ZMHB (collection Becker)] - labels: "Saloniki" / "26242"; "Zool. Mus." / "Berlin” [remarks: Becker catalogue: 26242: "*Alloconeurus lacustris* Scopoli, Saloniki, Türkei", deposited: 25. Mai 1890];

(**GR-05**) - **GREECE**: 1♀, Ionian Islands, Corfu, at old harbor, 29.iv.1885, leg. T. Becker [ZMHB (collection Becker)] - labels: "*Liancalus*" / "*lacustris*" / "4790" /[bottom side] "Corfu" / "29/4.85"; "Zool. Mus." / "Berlin" [remarks: Becker catalogue: 4790: "*Alloconeurus lacustris* Scopoli, 1♀, Corfu, Wiesen und Gärten am alten Hafen", deposited: 29. April 1885];

(**GR-06**) - **GREECE**: 1♀, Trikala, Pert(o)uli, Pindos (1150m), 4-12.x.1958, leg. Mannheims [ZMFK] - labels: Griechenland 1958 / Pindos 1150m Pertuli / 4.-12.X. Mannheims; [upside down] *Orthoceratium* ♀ / *lacustre* (Scopoli) / H. Ulrich det. 1983; [upside down] ZFMK DIP / 00019528;

(**GR-07**) - **GREECE**: 1♂, Serres, Neo Petritsi, Beles (Kerkini) Mts, 41°19'02.1"N, 23°12'05.0"E, 1485m, 8-14.ix.2008, MT, leg. Gordon Ramel [MAPC]; 1♀, same site, 15-21.ix.2008, MT, leg. Gordon Ramel [MAPC]; 1♀, same site, 22-28.ix.2008, MT, leg. Gordon Ramel [MAPC]; 1♂, same site, 30.vi-6.vii.2008, MT, leg. Gordon Ramel [MAPC] (all W);

(**GR-08**) - **GREECE**: 1♂, Serres, Vironia, Beles (Kerkini) Mts, 41°19'15.4"N, 23°13'39.6"E, 1150m, 26.v-1.vi.2008, MT, leg. Gordon Ramel [MAPC]; 5♀, same site, 7-13.vii.2008, MT, leg. Gordon Ramel [MAPC]; 3♂, 6♀, same site, 8-14.ix.2008, MT, leg. Gordon Ramel [MAPC]; 1♂, 4♀, same site, 14-20.vii.2008, MT, leg. Gordon Ramel [MAPC]; 1♂, 0♀, same site, 15-21.ix.2008, MT, leg. Gordon Ramel [MAPC]; 1♀, same site, 23-29.vi.2008, MT, leg. Gordon Ramel [MAPC]; 1♂, 5♀, same site, 28.vii-3.viii.2008, MT, leg. Gordon Ramel [MAPC]; 1♂, same site, 30.vi.2008-6.vii.2008, MT, leg. Gordon Ramel [MAPC]; 1♂, 1♀, same site, 1.viii.2008-7.ix.2008, MT, leg. Gordon Ramel [MAPC] (all W); 2♂, 3♀ from these samples deposited in RBINS;

(**IT-01**) - **ITALY**: 1♂, Sardinia, Iglesias, Marganai, Tintillonis, 22-25.ix.2004, leg. D. Birteli, P. Cerretti, E. Gatti, F. Mason & D. Whitmore [MAPC] - labels: "Sardegna (Cagliari), Iglesias, Marganai, Tintillonis, 480m, 22-25.ix.2004, radura con *Foeniculum vulgaris*, D. Birteli, P. Cerretti, E. Gatti, F. Mason, D. Whitmore leg, PS-CNBF" (W);

(**IT-02**) - **ITALY**: 1♂, Syracuse (in auton. region Sicilia), Syracuse, 23.iv, leg. T. Becker [ZMHB (collection Loew)] - labels: "Syrakus" /"23.4.Zeller"; "12671"; "Zool. Mus." / "Berlin" [remarks: Becker catalogue: 12671: "*Liancalus lacustris* Scopoli (*viridipes* Macq.), coll. H. Loew" (without date in the Diptera catalogue)]; 1♀, Syracuse (in auton. region Sicilia), Syracuse, 24.iv, leg. T. Becker [ZMHB (collection Loew)] - labels: "Syrakus" /"24.4.Zeller"; "12671"; "Zool. Mus." / "Berlin"; [remarks: Becker catalogue: 12671: "*Liancalus lacustris* Scopoli (*viridipes* Macq.), coll. H. Loew" (without date in the Diptera catalogue)]; 1♀, Syracuse (in auton. region Sicilia), Syracuse, 4.v, leg. T. Becker [ZMHB (collection Loew)] - labels: "Syrakus" /"4.5.Zeller"; "12671"; "Zool. Mus." / "Berlin" [remarks: Becker catalogue: 12671: "*Liancalus lacustris* Scopoli (*viridipes* Macq.), coll. H. Loew" (without date in the Diptera catalogue)]; 1♂, Syracuse (in auton. region Sicilia), Syracuse, leg. T. Becker [ZMHB (collection Becker)] - labels: "Syrakus" /"53924.7"; "Zool. Mus." / "Berlin" [remarks: Becker catalogue: 53924: "*Alloconeurus lacustris* Scopoli, 1♂, Syrakus, am Meeresstrande auf Blüten", deposited: 4. - 8. Mai 1906]; 1♂, Syracuse (in auton. region Sicilia), Syracuse [NHMW] - labels: "Siracusa" / "Sicil. Zerny" / "1.-4.V.'21"; 1♂, same locality [NHMW] - labels: "Siracusa" / "Sicil. Zerny" / "1.-4.V.'21"; 1♂, same locality [NHMW] - labels: "Siracusa" / "Sicil. Zerny" / "1.-4.V.'21"; 1♀, same locality [NHMW] - labels: "Siracusa" / "Sicil. Zerny" / "1.-4.V.'21";

(**IT-03**) - **ITALY**: 1♀, Veneto (region), Venice, Vignole, 22.x.1958 [BMNH] - labels: "Barene Vignole" / "22-X.58"; "*Orthoceratium*" / "*lacustre* Scop." / "det. L. Rampini"; barcode "NHMUK010627340"; 1♂, Veneto (region), Venice, Vignole, 22.x.1958 [BMNH] - labels: "Barene Vignole" / "22-X.58"; "*Orthoceratium*" / "*lacustre* Scop." / "det. L. Rampini"; barcode "NHMUK010627342"; 1♀, Veneto (region), Venice, 29.ix.1923, leg. G. Enderlein [ZMHB] - labels: [green rectangular] "29 9.23" / "Venedig" / "Lido" / "G. Enderlein G"; "*Orthoceratium*" / "*lacustre* Scop" / "O. Parent det."; 1♂, Veneto (region), Venice, 6.x.1960 [BMNH] - labels: "LAGUNA Veneta" / "Barene Val Doga" / "6-10-60"; "*Orthoceratium*" / "*lacustre* Scop." / "det. L. Rampini"; barcode "NHMUK010627341"; 1♀, Veneto (region), Venice [BMNH] - labels: "LAGUNA VENETA" / "Ricerche lagunari 1944-48" / "Staz. terr. N." / "Giordani Soika 108"; "108"; "*Orthoceratium*" / "*lacustre* Scop." / "det. L. Rampini"; barcode "NHMUK010627347"; 1♀, Veneto (region), Venice [NHMW] - labels: "Vineza" / "21/7.

1859"; "*lacustris*" / "det. Schiner"; 1♀, Veneto (region), Venice, Lido di Venezia [NHMW] - labels: "Lido" / "2,6,76"; "Coll. " / "Bgst."; "*lacustris*" / "♀ Scop.";

(IT-04) – see neotype specimen; 1♂, Gorizia (in region Friuli-Venezia Giulia), Görz [= Gorizia] [NHMW] - labels: "Görz" / "5.65"; "Mik" / "Illyria" / "Görz" / "5.65"; "*lacustris*" / "det. Mik"; 1♀, same locality [NHMW] - labels: "Görz" / "5.63"; "Mik" / "Illyria" / "Görz" / "5.65"; [rectangular grey] "♂"; "*lacustris*" / "det. Mik"; 1♀, same locality [NHMW] - labels: "Görz" / "5.65"; "Mik" / "Illyria" / "Görz" / "5.65"; "*lacustris*" / "det. Mik";

(IT-05) - ITALY: 1♀, Taranto (in region Apulia), Taranto, 1.v.1918, leg. E. Hargreaves [BMNH] - labels: "Pres. by" / "Imp. Bur. Ent." / "Brit. Mus." / "1927-237."; "*Orthoceratium*" / "*lacustre* Scop" / "O. Parent"; "ITALY" / "TARANTO" / "1-V.1918" / "E. HARGREAVES"; barcode "NHMUK010627343";

(IT-06) - ITALY: 1♂, Gorizia (in region Friuli-Venezia Giulia), Gulf of Trieste, [Castello?] Miramare [NHMW] - labels: "Miramare" / "15.5.64"; "Mik"/ "Illyria"; "*lacustris*" / "det. Mik";

(IT-07) - ITALY: 1♂, Gorizia (in region Friuli-Venezia Giulia), Triest [NHMW] - labels: "30.5.87" / "Handl."; "Triest" [remarks: right cxl with stark black bristle at basal 1/4]; 1♂, same locality [NHMW] - labels: "30.5.87" / "Handl."; "Triest"; 1♂, same locality [NHMW] - labels: "30.5.87" / "Handl."; "Triest"; 1♂, same data - labels: "30.5.87" / "Handl."; "Triest"; 1♀, same locality [NHMW] - labels: [white square] "487"; "Coll. " / "Bgst."; "Triest." / "*Liancalus*" / "*lacustris*" / "♀ Scop."; "*lacustris* ♀" / "det. Bergenst."; 1♀, same locality [NHMW] - labels: "29.5.87" / "Handl. Ant."; "Triest"; 1♀, same locality [NHMW] - labels: "30.5.87" / "Handl."; "Triest"; 1♀, same locality [NHMW] - labels: "30.5.87" / "Handl."; "Triest";

(IT-08) - ITALY: 1♂, Livorno [NHMW] - labels: [white square] "236"; "Livorno"; "*lacustris*" / "Alte Sammlung";

(ME-01) - MONTENEGRO: 1♀, Central Region, Cetinje, 21.v.1889, leg. T. Becker [ZMHB (collection Becker)] - labels: [brownish grey rectangular] "Dalmatien" / "21/5 22809"; "*Alloconeurus*" / "*lacustris*" / "Scop."; "Zool. Mus." / "Berlin" [remarks: Becker catalogue: 22809: "*Alloconeurus lacustris* Scopoli, 1♀, Cetinje, Montenegro", deposited: 21. Mai 1889];

Three *Orthoceratium lacustre* specimens had only the following labels: 1♀ [ZMHB (collection Duda)] - labels: [white square] "*Orthocera-*" / "*tium*" / "*lacustre*" / "Scop ♀ dg"; "Zool. Mus." / "Berlin"; 1♂ [ZMHB] - labels: "Zool. Mus." / "Berlin"; 1♀ [ZMHB] - labels: "Zool. Mus." / "Berlin".

Orthoceratium sabulosum (Becker, 1907)

(DZ-01) - ALGERIA: 1♂, Oran, Misserghine, 1929, leg. Alluaud & Jeannel [MNHN (collection générale)] - labels: [green rectangular] "MUSEUM PARIS" / "ALGERIE" / "MISSERGHINE" / "Alluaud et Jeannel 1929"; "*Orthoceratium* ♂" / "*lacustre* Scop " / "O. Parent";

(DZ-02) - ALGERIA: 1♂, Algiers, La Croix [= Vieux Kouba], 1.ix.1913 [ZMHB] - labels: "Algeria" / "LaCroix" /[bottom side] "1913" / "IX. 1."; "Zool. Mus." / "Berlin"; 1♂, same site [ZMHB] - labels: "Algeria" / "LaCroix" /[bottom side] "1913" / "IX. 1."; "Zool. Mus." / "Berlin";

(DZ-03) - ALGERIA: 1♂, El Tarf, La Calle [= El Kala], 28.ix.1913 [ZMHB] - labels: "Tunisia" / "LaCalle" /[bottom side] "1913" / "IX. 28."; "Zool. Mus." / "Berlin";

(BE-01) - BELGIUM: 1♂, West-Vlaanderen, Knokke, Het Zwin NR, 9.ix.1931 [MNHN (collection générale)] - labels: "Zwyn" / "9.IX.31"; "*O. lacustre*" / "Schr"; 1♂, same site, 9.ix.1931 [MNHN (collection Parent)] - labels: "Zwyn" / "9.IX.31"; 1♂, same site, 9.ix.1931 [MNHN (collection Parent)] - labels: "Zwyn" / "9.IX.31"; 1♀, same site, 9.ix.1931 [MNHN (collection Parent)] - labels: "Zwyn" / "9.IX.31"; 1♀, same site, 9.ix.1931 [MNHN (collection Parent)] - labels: "Zwyn" / "9.IX.31"; 1♀, same site, 9.ix.1931 [MNHN (collection Parent)] - labels: "Zwyn" / "9.IX.31"; 1♀, same site, 9.ix.1931 [MNHN (collection Parent)] - labels: "Zwyn" / "9.IX.31"; 1♀, same site, 9.ix.1931 [RBINS] - labels: "♀"; "Zwyn" / "9.IX.31"; "R. Mus. Hist. Nat." / "Belg. I.G. 11.275"; "*Orthoceratium*" / "*lacustre* (Scop). ♀" / "O. PARENT det"; 1♂, same site, 9.ix.1931 [RBINS] - labels: "♂"; "Zwyn" / "9.IX.31"; "R. Mus. Hist. Nat." / "Belg. I.G. 11.275"; "*Orthoceratium*" / "*lacustre* (Scop). ♂" / "O. PARENT det"; 1♂, 3♀, same site, 21.vi.1936, leg. M. Goetghebuer [IRSNB]; 1♂, same site, 16.vi.1939 [IRSNB]; 1♂, same site, 16.vi.1939 [RBINS] - labels: "Zwyn" / "16.VI.1939"; "*Orthoceratium* ♂" / "*lacustre* (Scop.)" / "det. H.G.J. Meuffels"; 1♂, same site, 3.vii.1955, leg. L. Marnef [IRSNB]; 1♂, same site, 3.vii.1955, leg. L. Marnef [RBINS] - labels: "Knokke" / "Zwin" / "3.VII.1955"; "Ex. Coll." / "L. Marnef"; "*Orthoceratium* ♂" / "*lacustre* (Scop.)" / "H. Meuffels det., 1986"; 2♀, same site, 8.vii.1978 [coll. Gembloux]; 1♂, same site, 21.viii.1987, leg. M. Pottier [RBINS] - labels: "B. Knokke" / "21-VIII-1987" / "ZWIN" / "leg. M. Pottier"; "Ch.Verbeke Det. 1987" / "♂ *ORTHOCERATIUM*" / "*LACUSTRE* Scop."; 6♂, 16♀, same site, 51°21'44.0"N, 3°21'34.7"E, 22.ix.2016, SW, leg. Marc Pollet [MAPC] (W); 7♂, 6♀, same site, 22.ix.2016, SW, leg. Anja De Braekeleer [MAPC] (W);

(BE-02) - BELGIUM: 1♂, West-Vlaanderen, Knokke-aan-Zee, 5.ix.1921, leg. M. Goetghebuer [IRSNB] - [see Goetghebuer 1934]; 1♂, 7♀, same site, 25.ix.1921, leg. M. Goetghebuer [IRSNB]; 2♂, 3♀, same site, 14.ix.1924, leg. M. Goetghebuer [IRSNB]; 1♂, 1♀, same site, 17.ix.1924, leg. M. Goetghebuer [IRSNB]; 1♂, same site, 7.ix.1955, leg. M. Bequaert [IRSNB]; 1♀, same site, 17.x.1969, leg. Jean Verbeke [IRSNB]; 1♂, West-Vlaanderen, Knokke-Heist, 14.ix.1923 [MNHN (collection Parent)] - labels: "Knocke s/m." / "14-9-23"; "*O. lacustre*" / "Scop"; 1♀, same site, 14.ix.1923 [MNHN (collection Parent)] - labels: "Knocke s/m." / "14-9-23"; "*O. lacustre*" / "Scop"; 1♀, same site, 17.x.1969, leg. J. Verbeke [RBINS] - labels: "(B) - W.VL" / "Knokke a-z" / "17-X-1969" / "J. Verbeke"; "R. I. Sc. N. B." / "I. G. 23.854"; "*Orthoceratium* ♀" / "*lacustre* (Scop.)" / "det. H.G.J. Meuffels";

(BE-03) - BELGIUM: 7♂, 8♀, West-Vlaanderen, Dudzele, along southern part of Zeevaartstraat, 51°17'6.75"N, 3°12'39.24"E, 22.ix.2016, SW, leg. Marc Pollet [MAPC] (W); 14♂, 16♀, same data, leg. Anja De Braekeleer [MAPC] (W); 2♂, 3f # from these samples deposited in RBINS;

(BE-04) - BELGIUM: 2♂, 1♀, West-Vlaanderen, Dudzele, Dudzele Noord, 51°18'30.60"N, 3°12'17.60"E, 19.x.2012, YPT, leg. Frank Van de Meutter [MAPC] (W);

(**BE-05**) - **BELGIUM**: 3♂, 7♀, West-Vlaanderen, Lissewege, Monikkenwerve NR, 51°17'22.62"N, 3°12'19.49"E, 22.ix.2016, SW, leg. Marc Pollet [MAPC] (W); 3♂, 12♀, same data, leg. Anja De Braekeleer [MAPC] (W);

(**DK-01**) - **DENMARK**: 1♂, 1♀, South Jutland, Skallingen, leg. Eric Rald [ZMUC, see also Grichanov 2010)];

(**FR-02**) - **FRANCE**: 1♂, Morbihan (dept.), Vannes, 28.vi.1922 [MNHN (collection générale)] - labels: "Vannes" / "28.VI.22"; "*Orthoceratium*" / "*lacustre* Scop " / "det. O Parent";

(**DE-01**) - **GERMANY**: 1♂, Niedersachsen (Land), Borkum Island, vii.1895, leg. B. Lichtwardt [MNHN (collection Parent)] - labels: "Borkum." / "VII.95"; "coll. Lichtwardt"; "*Orthocerat*." / "*lacustre* Scop" / "27.03 (?)"; "*O. lacustre*" / "Scop"; 1♀, same data [MNHN (collection Parent)] - labels: "Borkum." / "VII.95"; "coll. Lichtwardt"; "*Orthocerat*." / "*lacustre* Scop" / "Dr. Licht."; "*O. lacustre*" / "Scop";

(**GB-01**) - **GREAT BRITAIN**: 1♀, Suffolk, Aldeburgh, 19.ix.1900, leg. Verrall Bequest [BMNH] - labels: "Verrall Bequest." / "B.M. 1911-411." / "*Alloeoneurus*" / "*lacustris*. Scop"; [white circular] "Aldeburgh" / "19/9/00" / "ColY"; barcode "NHMUK010627380"; 1♂, same site, 27.viii.1907, leg. Verrall Bequest [BMNH] - labels: "Verrall Bequest." / "B.M. 1911-411." / "*Alloeoneurus*" / "*lacustris*. Scop"; [white circular] "Aldeburgh" / "12/9/07"; barcode "NHMUK010627378"; 1♂, same site, 15.ix.1907, leg. Lt.-Col. Yerbury [BMNH] - labels: "Aldeburgh" / "Suffolk" / "15.IX.1907" / "Lt. Col. Yerbury"; barcode "NHMUK010627389"; 1♂, same site, 15.ix.1907, leg. Lt.-Col. Yerbury [BMNH] - labels: "Aldeburgh" / "Suffolk" / "15.IX.1907" / "Lt. Col. Yerbury"; barcode "NHMUK010627392"; 1♀, same site, 15.ix.1907, leg. Lt.-Col. Yerbury [BMNH] - labels: "Aldeburgh" / "Suffolk" / "15.IX.1907" / "Lt. Col. Yerbury"; barcode "NHMUK010627391"; 1♂, same site, 15.ix.1907, leg. Lt.-Col. Yerbury [BMNH] - labels: "Aldeburgh" / "Suffolk" / "15.IX.1907" / "Lt. Col. Yerbury"; barcode "NHMUK010627394"; 1♀, same site, 17.ix.1907, leg. Verrall Bequest [BMNH] - labels: "Verrall Bequest." / "B.M. 1911-411." / "*Alloeoneurus*" / "*lacustris*. Scop"; [white circular] "Aldeburgh" / "17/9/07"; barcode "NHMUK010627368"; 1♀, same site, 17.vi.1965, [BMNH] - labels: "*L. virens*" / "Aldeburgh" / "17.6.65"; [white rectangular, upside down] "Pres. By" / "C.H. Andrewes" / "BMNH 1987-97"; barcode "NHMUK010627379";

(**GB-02**) - **GREAT BRITAIN**: 1♂, Dorset, Arne, 18.ix.1955, leg. E.A. Fonseca [BMNH] - labels: "*Orthoceratium*" / "*lacustre*" / "Arne" / "Dorset" / "18.9.55"; "Pres. by Sir C.H. Andrews" / "B.M. 1978-80"; barcode "NHMUK010627370";

(**GB-03**) - **GREAT BRITAIN**: 1♂, Kent, Berengrave Lane LNR, TQ 8267, 14.viii.1983, leg. Laurence Clemons [see Clemons 2003)]; same site, 6.vii.1994, leg. Laurence Clemons [see Clemons 2003];

(**GB-04**) - **GREAT BRITAIN**: 1♀, North Somerset, Berrow, 10.vi.1950, leg. E.A. Fonseca [BMNH] - labels: [white square] "Berrow" / "Som. N." / "10.vi.50" / "E.A. Fonseca"; [white rectangular, upside down] "Pres. by E.C.M." / "Assis Fonseca" / "BMNH 1988-212"; barcode "NHMUK010627355";

(**GB-05**) - **GREAT BRITAIN**: 2♂, Kent, Conyer old brickworks, TQ 962652, 23.viii.2001, leg. Laurence Clemons [see Clemons 2003];

(**GB-06**) - **GREAT BRITAIN**: Kent, Darland Banks, TQ 793655, 27.vi.1984, leg. Laurence Clemons [see Clemons 2003];

(**GB-07**) - **GREAT BRITAIN**: 1♂, Kent, Gravesend, 8.ix.1907, leg. Lt.-Col. Yerbury [BMNH] - labels: "Gravesend," / "Kent" / "8.IX.1907" / "Lt. Col. Yerbury"; barcode "NHMUK010627386"; 1♀, Kent, Gravesend, 11.ix.1907, leg. Lt.-Col. Yerbury [BMNH] - labels: "Gravesend," / "Kent" / "11.IX.1907" / "Lt. Col. Yerbury"; barcode "NHMUK010627393";

(**GB-08**) - **GREAT BRITAIN**: 1♂, Kent, Great Chattenden Wood, TQ 747734, 1.vi.2002, leg. Laurence Clemons [see Clemons 2003];

(**GB-09**) - **GREAT BRITAIN**: 1♂, Kent, Kingsnorth-on-Hoo, TQ 812736, 5.vii.1987, leg. Laurence Clemons [see Clemons 2003]; 1♀, same site, 13.vi.1992, leg. Laurence Clemons [see Clemons 2003];

(**GB-10**) - **GREAT BRITAIN**: 1♂, Cornwall, Lelant, 24.viii.1912, leg. Lt.-Col. Yerbury [BMNH] - labels: "Lelant," / "Cornwall." / "24.VIII.1912" / "Lt.-Col. Yerbury" / "1913-449."; barcode "NHMUK010627369"; 1♂, same site, 24.viii.1912, leg. Lt.-Col. Yerbury [BMNH] - labels: "Lelant," / "Cornwall." / "24.VIII.1912" / "Lt.-Col. Yerbury" / "1913-449."; barcode "NHMUK010627388";

(**GB-11**) - **GREAT BRITAIN**: 1♂, Dorset, Lodmoor, 1.x.1949, leg. E.A. Fonseca [BMNH] - labels: [white square] "Lodmoor" / "Dorset" / "1.x.49" / "E.A. Fonseca"; [white rectangular, upside down] "Pres. by E.C.M." / "Assis Fonseca" / "BMNH 1988-212"; barcode "NHMUK010627352"; 1♂, same site, 1.x.1949, leg. E.A. Fonseca [BMNH] - labels: [white square] "Lodmoor" / "Dorset" / "1.x.49" / "E.A. Fonseca"; [white rectangular, upside down] "Pres. by E.C.M." / "Assis Fonseca" / "BMNH 1988-212"; barcode "NHMUK010627350"; 1♂, same site, 1.x.1949, leg. E.A. Fonseca [BMNH] - labels: [white square] "Lodmoor" / "Dorset" / "1.x.49" / "E.A. Fonseca"; [white rectangular, upside down] "Pres. by E.C.M." / "Assis Fonseca" / "BMNH 1988-212"; barcode "NHMUK010627357"; 1♀, same site, 1.x.1949, leg. E.A. Fonseca [BMNH] - labels: [white square] "Lodmoor" / "Dorset" / "1.x.49" / "E.A. Fonseca"; [white rectangular, upside down] "Pres. by E.C.M." / "Assis Fonseca" / "BMNH 1988-212"; barcode "NHMUK010627354"; 1♀, same site, 1.x.1949, leg. E.A. Fonseca [BMNH] - labels: [white square] "Lodmoor" / "Dorset" / "1.x.49" / "E.A. Fonseca"; [white rectangular, upside down] "Pres. by E.C.M." / "Assis Fonseca" / "BMNH 1988-212"; barcode "NHMUK010627349";

(**GB-12**) - **GREAT BRITAIN**: Kent, Maiden Lane allotment site Crayford, TQ 526749, 30.viii.1993, leg. Laurence Clemons [see Clemons 2003];

(**GB-13**) - **GREAT BRITAIN**: 1♀, Glamorganshire, Merthyr Mawr, 16.vi.1992, leg. C.E. Dyte [BMNH] - labels: "WALES:" / "Glamorgan," / "Merthyr Mawr." / "16.vi.1992" / "C.E. Dyte"; "*Ortoceratium* ♀" / "*lacustre* (Scop)" / "det. C.E.Dyte 1992"; barcode "NHMUK010627317";

(**GB-14**) - **GREAT BRITAIN**: Kent, Murston, TQ 922646, 17.vii.1981, leg. Laurence Clemons [see Clemons 2003]; 1♀, same location, TQ 929651, 8.vi.1982, leg. Laurence Clemons [see Clemons 2003]; 1♀, same location, TQ 925661, 5.ix.1982, leg. Laurence [see Clemons 2003]; 1♂, 1♀, same location, TQ 928648, 16.ix.1983, leg. Laurence Clemons [see Clemons 2003];

(**GB-15**) - **GREAT BRITAIN**: 1♂, Kent, Oare Gravel Pits, TR 003623, 21.vii.1983, leg. Laurence Clemons [see Clemons 2003)];

(**GB-16**) - **GREAT BRITAIN**: 1♂, Glamorganshire (Gower), Oxwich, 26.vi.1952, leg. J. Cowley [BMNH] - labels: "Oxwich, Gower" / "Glamorgan" / "J. Cowley" / "26.VI.1952"; [upside down] "J.CowleyColl." / "B.M. 1968-70"; barcode "NHMUK010627365"; (GB-16) - GREAT BRITAIN: 1♂, same site, 10-16.vi.1955, leg. E.A. Fonseca [BMNH] - labels: [white square] "Oxwich" / "Gower Glam." / "10-16.vi.55" / "E.A. Fonseca"; [upside down] "Pres. by E.C.M." / "Assis Fonseca" / "BMNH 1988-212"; barcode "NHMUK010627351"; 1♂, same site, 25.vi.1956, leg. E.A. Fonseca [BMNH] - labels: [white square] "Oxwich" / "Gower Glam." / "25.vi.56" / "E.A. Fonseca"; [upside down] "Pres. by E.C.M." / "Assis Fonseca" / "BMNH 1988-212"; barcode "NHMUK010627356"; [remarks:]; 1♂, same site, 26.vi.1956, leg. E.A. Fonseca [BMNH] - labels: [white square] "Oxwich" / "Gower Glam." / "25.vi.56" / "E.A. Fonseca"; [upside down] "Pres. by E.C.M." / "Assis Fonseca" / "BMNH 1988-212"; barcode "NHMUK010627353";

(**GB-17**) - **GREAT BRITAIN**: 1♂, Essex, Parkeston, 3.ix.1910, leg. Lt.-Col. Yerbury [BMNH] - labels: "Parkstone," / "Essex" / "3.IX.1910." / "Lt.-Col. Yerbury" / "1911-121."; barcode "NHMUK010627376" [remarks: "Parkstone" on label];

(**GB-18**) - **GREAT BRITAIN**: 1♀, Glamorganshire, Porthcawl, 23.vi.1903, leg. Lt.-Col. Yerbury [BMNH] - labels: "Porthcawl," / "Glamorganshire, " / "S. Wales" / "23.VI.1903" / "Lt. Col. Yerbury"; barcode "NHMUK010627387"; 1♂, same site, 28.vi.1903, leg. Lt.-Col. Yerbury [BMNH] - labels: "Porthcawl," / "Glamorganshire, " / "S. Wales" / "28.VI.1903" / "Lt. Col. Yerbury"; barcode "NHMUK010627382" [remarks: head lacking]; 1♂, same site, 7.vii.1906, leg. Lt.-Col. Yerbury [BMNH] - labels: "Porthcawl," / "Glamorganshire, " / "S. Wales" / "7.VII.1906" / "Lt. Col. Yerbury" / "1907-101"; barcode "NHMUK010627390"; 1♀, same site, 7.vii.1906, leg. Lt.-Col. Yerbury [BMNH] - labels: "Porthcawl," / "Glamorganshire, " / "S. Wales" / "7.VII.1906" / "Lt. Col. Yerbury" / "1907-101"; barcode "NHMUK010627384"; 1♂, same site, 12.vii.1906, leg. Lt.-Col. Yerbury [BMNH] - labels: "Porthcawl," / "Glamorganshire, " / "S. Wales" / "12.VII.1906" / "Lt. Col. Yerbury" / "1907-101"; barcode "NHMUK010627385"; 1♀, same site, 12.vii.1906, leg. Lt.-Col. Yerbury [BMNH] - labels: "Porthcawl," / "Glamorganshire, " / "S. Wales" / "12.VII.1906" / "Lt. Col. Yerbury" / "1907-101"; barcode "NHMUK010627381" [remarks: anterior and posterior body parts separated]; 1♀, same site, 12.vii.1906, leg. Verrall Bequest [BMNH] - labels: "Verrall Bequest." / "B.M. 1911-411." / "*Alloeoneurus*" / "*lacustris*. Scop"; [white circular] "Porthcawl"/ "12/7/06" / "ColY"; barcode "NHMUK010627374";

(**GB-19**) - **GREAT BRITAIN**: 1♂, Suffolk, River Deben, 27.viii.1907, leg. Verrall Bequest [BMNH] - labels: "Verrall Bequest." / "B.M. 1911-411." / "*Alloeoneurus*" / "*lacustris*. Scop"; [white circular] "R Deben" / "28/8/07"; barcode "NHMUK010627375";

(**GB-20**) - **GREAT BRITAIN**: 1♀, Norfolk, Salthouse, 26.ix.1957 [BMNH] - labels: "*Liancalus*" / "♀" / "Salthouse" / "26.9.57"; [upside down] "Pres. By" / "C.H. Andrewes" / "BMNH 1987-97"; barcode "NHMUK010627366"; 1♂, same site, 26.ix.1957 [BMNH] - labels: "*Liancalus*" / "♂" / "Salthouse" / "26.9.57"; [upside down] "Pres. By" / "C.H. Andrewes" / "BMNH 1987-97"; barcode "NHMUK010627377"; 1♀, same site, 26.ix.1957 [BMNH] - labels: "*Liancalus*" / "♀" / "Salthouse" / "26.9.57"; [white rectangular, upside down] "Pres. By" / "C.H. Andrewes" / "BMNH 1987-97"; barcode "NHMUK010627359";

(**GB-21**) - **GREAT BRITAIN**: 1♀, Kent, Sheppey,Windmill Creek, 12.x.1969, leg. R.I. Vane-Wright [BMNH] - labels: "ENGLAND: Kent." / "Sheppey" / "Windmill Creek" / "12.x.1969" / "R.I. Vane-Wright." / "TQ 963 691"; "*Orthoceratium*" / "*lacustre* Scop. ♀" / "det: E.A. Fonseca"; barcode "NHMUK010627362";

(**GB-22**) - **GREAT BRITAIN**: 1♂, Kent, Sheppey, 11.x.1969, leg. R.I. Vane-Wright [BMNH] - labels: "ENGLAND: Kent." / "Sheppey" / "11.x.1969" / "TQ 914 698" / "R.I. Vane-Wright."; "*Orthoceratium*" / "*lacustre* Scop. ♂" / "det: E.A. Fonseca"; barcode "NHMUK010627373";

(**GB-23**) - **GREAT BRITAIN**: Dorset, Studland, 4.ix.1910, leg. Lt.-Col. Yerbury [BMNH] - labels: "Studland," / "Dorset." / "4.IX.1910" / "Lt.-Col. Yerbury" / "1911-121."; barcode "NHMUK010627383" [remarks: only one wing left];

(**GB-24**) - **GREAT BRITAIN**: 2♀, Kent, Swalecliffe, TR 139677, 23.x.1983, leg. Laurence Clemons [see Clemons 2003];

(**GB-25**) - **GREAT BRITAIN**: 1♂, 1♀, Kent, Mill House near Tonge, TQ 932635, 18.vii.1983, leg. Laurence Clemons [see Clemons 2003];

(**GB-26**) - **GREAT BRITAIN**: 1♀, Glamorganshire (Gower), Whiteford, 19.viii.1971, leg. [BMNH] - labels: "*O. lacustre*" / "Whiteford ♀" / "Gower 19.8.71"; "Pres. by Sir C.H. Andrews" / "B.M. 1978-80"; barcode "NHMUK010627372"; 1♂, same site, 21.viii.1971, leg. [BMNH] - labels: "*O. lacustre*" / "Whiteford ♂" / "Gower 21.8.71"; "Pres. by Sir C.H. Andrews" / "B.M. 1978-80"; barcode "NHMUK010627361";

(**GB-27**) - **GREAT BRITAIN**: 1♀, Glamorganshire, Bridgend, 6.viii.1908, leg. [BMNH] - labels: [white circular] "Bridgend" / "6.8.08"; barcode "NHMUK010627363";

(**GB-28**) - **GREAT BRITAIN**: 1♂, Norfolk, Holkham, coastal nature reserve, 2.x.1957 [BMNH] - labels: "*Liancalus*" / "♂" / "Holkham" / "2.x.57"; [upside down] "Pres. By" / "C.H. Andrewes" / "BMNH 1987-97"; barcode "NHMUK010627364"; 1♀, same site, 2.x.1957 [BMNH] - labels: "*Liancalus*" / "♂" /

"Holkham" / "2.x.57"; [upside down] "Pres. By" / "C.H. Andrewes" / "BMNH 1987-97"; barcode "NHMUK010627367"; 1♂, same site, 2.x.1957 [BMNH] - labels: "*Liancalus*" / "♂" / "Holkham" / "2.x.57"; [upside down] "Pres. By" / "C.H. Andrewes" / "BMNH 1987-97"; barcode "NHMUK010627358"; 1♀, same site, 2.x.1957 [BMNH] - labels: "*Liancalus*" / "♂" / "Holkham" / "2.x.57"; [white rectangular, upside down] "Pres. By" / "C.H. Andrewes" / "BMNH 1987-97"; barcode "NHMUK010627371";

(GB-29) - GREAT BRITAIN: 1♂, Suffolk, Boyton, 27.viii.1907, leg. Verrall Bequest [BMNH] - labels: "Verrall Bequest." / "B.M. 1911-411." / "*Alloeoneurus*" / "*lacustris*. Scop"; [white circular] "Boyton" / "27.8.07"; barcode "NHMUK010627360";

(GR-01) - GREECE: 1♀, North Aegean Islands, Lesvos, 1 km W Skala Kalloni (saltmarsh along West River), 17.v.2000, SW, leg. Marc Pollet [MAPC];

(GR-09) - GREECE: 1♂, North Aegean Islands, Lesvos, 10 km E of Skala Kalloni, 25.v.1995, leg. C.E. Dyte [BMNH] - labels: "saltmarsh 10 km E." / "of Skala Kallonis"; "GREECE, Lesvos:" / "25.v.1995 C.E.Dyte"; "BMNH(E) 2013-47" / " C.E. Dyte collection"; barcode "NHMUK010627335";

(GR-10) - GREECE: 1♀, North Aegean Islands, Lesvos, 2 km W of Skala Kalloni, 19.v.1995, leg. C.E. Dyte [BMNH] - labels: "saltmarsh river" / "mouth 2 km W of" / "Skala Kallonis"; "GREECE, Lesvos:" / "19.v.1995 C.E.Dyte"; "BMNH(E) 2013-47" / " C.E. Dyte collection"; barcode "NHMUK010627331"; 1♂, same data [BMNH] - labels: "saltmarsh river" / "mouth 2 km W of" / "Skala Kallonis"; "GREECE, Lesvos:" / "19.v.1995 C.E.Dyte"; "BMNH(E) 2013-47" / " C.E. Dyte collection"; barcode "NHMUK010627339"; 1♂, same data [BMNH] - labels: "saltmarsh river" / "mouth 2 km W of" / "Skala Kallonis"; "GREECE, Lesvos:" / "19.v.1995 C.E.Dyte"; "BMNH(E) 2013-47" / " C.E. Dyte collection"; barcode "NHMUK010627333"; 1♀, same data [BMNH] - labels: "saltmarsh river" / "mouth 2 km W of" / "Skala Kallonis"; "GREECE, Lesvos:" / "19.v.1995 C.E.Dyte"; "BMNH(E) 2013-47" / " C.E. Dyte collection"; barcode "NHMUK010627336"; 1♀, same data [BMNH] - labels: "saltmarsh river" / "mouth 2 km W of" / "Skala Kallonis"; "GREECE, Lesvos:" / "19.v.1995 C.E.Dyte"; "BMNH(E) 2013-47" / " C.E. Dyte collection"; barcode "NHMUK010627332"; 1♀, same data [BMNH] - labels: "saltmarsh river" / "mouth 2 km W of" / "Skala Kallonis"; "GREECE, Lesvos:" / "19.v.1995 C.E.Dyte"; "BMNH(E) 2013-47" / " C.E. Dyte collection"; barcode "NHMUK010627334"; 1♂, 1♀, same data [MAPC] - labels: "saltmarsh river" / "mouth 2 km W of" / "Skala Kallonis"; "GREECE, Lesvos:" / "19.v.1995 C.E.Dyte";

(GR-11) - GREECE: 1♀, Attica, Attica, 9.xii.1872 [MLUH] - labels: "Attica 9/12 72";

(IR-01) - IRAN: 2♂, East Azerbaijan, Chichakli (Forestry area), 38° 34.167' N, 46°30.091' E, 1,907m, 15.vi.2013, leg. Samad Khaghaninia [see Kazerani et al. 2014];

(IR-02) - IRAN: 2♂, East Azerbaijan, Keleyber (Forestry area), 38°51.548' N, 46°59.007' E, 1,783m, 10.vii.2013, leg. Samad Khaghaninia [see Kazerani et al. 2014];

(IT-09) - ITALY: 1♂, Sardinia, Iglesias [NHMW] - labels: "Iglesias. 5."; "7"; "Mik" / "Sardinia"; "*Alloenoneurus* ♂" / "*lacustris* Scop.";

(NL-01) - NETHERLANDS: 1♂, Zeeland, Yerseke,Yerseke Moer centraal, 22.ix.2012, leg. Niels-Jan Dek [MAPC] - labels: "*Orthoceratium lacustre* ♂" / "03-X-2012" / "51.49426, 4.01104";

(NL-02) - NETHERLANDS: 1♂, Zeeland, Cadzand, 15.ix.1989, leg. Bob van Aartsen [Kees de Kraker - ZMA Collection]; 1♂, same locality, 15.ix.1989-20.ix.1989, leg. Bob van Aartsen [RMNH] - labels: "Cadzand" / "15-20.IX.1989" / "B. v. Aartsen"; "*Orthoceratium* ♂" / "*lacustre* (Scop.)" / "det. H. Meuffels"; "ZMA" / "NL";

(NL-03) - NETHERLANDS: Zeeland, Cadzand, Het Zwin, 18.ix.1990, leg. Bob van Aartsen [Elias de Bree - ZMA Collection]; 2♀, same location, 18.ix.1990, leg. Bob van Aartsen [Elias de Bree - ZMA Collection]; 1♂, 2♀, same location, 18.ix.1990, leg. Bob van Aartsen [MEUFFELSPC]; 1♂, same location, 18.ix.1990, leg. Bob van Aartsen [VANAARTSENPC]; 1♂, same location, 18.ix.1990, leg. Bob van Aartsen [RMNH] - labels: "NETHERLANDS" / "Het Zwin" / "18.IX.1990" / "B. van Aartsen"; "*Orthoceratium* ♂" / "*lacustre* (Scop.)" / "det. H. Meuffels"; "ZMA" / "NL"; 1♀, same location, 18.ix.1990, leg. Bob van Aartsen [RMNH] - labels: "NETHERLANDS" / "Het Zwin" / "18.IX.1990" / "B. van Aartsen"; "*Orthoceratium* ♀" / "*lacustre* (Scop.)" / "det. H. Meuffels"; "ZMA" / "NL"; 1♂, same location, 18.ix.1990, leg. Bob van Aartsen [RMNH] - labels: "NETHERLANDS" / "Het Zwin" / "18.IX.1990" / "B. van Aartsen"; "*Orthoceratium* ♂" / "*lacustre* (Scop.)" / "det. H. Meuffels"; "ZMA" / "NL"; 1♀, same location, 18.ix.1990, leg. Bob van Aartsen [RMNH] - labels: "NETHERLANDS" / "Het Zwin" / "18.IX.1990" / "B. van Aartsen"; "*Orthoceratium* ♀" / "*lacustre* (Scop.)" / "det. H. Meuffels"; "ZMA" / "NL"; 1♂, same location, 0♀, 18.ix.1990, leg. Bob van Aartsen [RMNH] - labels: "NETHERLANDS" / "Het Zwin" / "18.IX.1990" / "B. van Aartsen"; "ZMA" / "NL";

(NL-04) - NETHERLANDS: 1♀, Friesland, Ameland, duinen N van vliegveld, 19.vi.1970, leg. M.J. Delfos [RML];

(NL-05) - NETHERLANDS: 1♂, Friesland, Terschelling, 22.viii.1977, leg. W. van Vierssen c.s. [RMNH] - labels: "TSbs" / "22081977"; "Terschelling" / "W.v. Vierssen" / "c.s."; "*Orthoceratium* ♂" / "*lacustre* (Scop.)" / "det. Meuffels" / "12852"; "ZMA" / "NL" [see Meuffels 1981];

(NL-06) - NETHERLANDS: 1♂, Zuid-Holland, Ouddorp, 9.x.1990, leg. Bob van Aartsen [RMNH] - labels: "NETHERLANDS" / "Ouddorp" / "9.X.1990" / "B. van Aartsen"; "*Orthoceratium* ♂" / "*lacustre* (Scop.)" / "det. H. Meuffels"; "ZMA" / "NL"; 1♀, same locality, 15.ix.1992, leg. Bob van Aartsen [RMNH] - labels: "NEDERLAND" / "Ouddorp" / "15.IX.1992" / "BvAartsen"; "*Orthoceratium* ♀" / "*lacustre* (Scop.)" / "det. H. Meuffels"; "ZMA" / "NL"; 1♀, same locality, 15.ix.1992, leg. Bob van Aartsen [RMNH] - labels: "NEDERLAND" / "Ouddorp" / "15.IX.1992" / "BvAartsen"; "ZMA" / "NL"; 1♀, same locality, 15.ix.1992, leg. Bob van Aartsen [RMNH] - labels: "NEDERLAND" / "Ouddorp" / "15.IX.1992" / "BvAartsen"; "ZMA" / "NL"; 1♀, same locality, 15.ix.1992, leg. Bob van Aartsen [RMNH] - labels: "NEDERLAND" / "Ouddorp" / "15.IX.1992" / "BvAartsen"; "ZMA" / "NL"; 1♂, same locality, 15.ix.1992, leg. Bob van Aartsen [RMNH] - labels: "NEDERLAND" / "Ouddorp" / "15.IX.1992" / "BvAartsen";

"*Orthoceratium* ♂" / "*lacustre* (Scop.)" / "det. H. Meuffels"; "ZMA" / "NL"; 1♀, same locality, 16.ix.1992, leg. Bob van Aartsen [RMNH] - labels: "NEDERLAND" / "Ouddorp" / "16.IX.1992" / "BvAartsen"; "*Orthoceratium* ♀" / "*lacustre* (Scop.)" / "det. H. Meuffels"; "ZMA" / "NL";

(PT-01) - PORTUGAL: 2♂, Beira Litoral, Lousã, Lousã, 40°04'28.6"N, 8°12'41.9"W, 700m, 20.vii.2011, SW, leg. Rui Andrade [MAPC] (W);

(PT-02) - PORTUGAL: 1♂, Douro Litoral, Canidelo, Vila Nova de Gaia, Reserva Natural Local do Estuário do Douro (Cabedelo), 41°08'13.3"N, 8°39'53.4"W, 7,5m, 14.x.2010, SW, leg. Rui Andrade [IRSNB] (W);

(PT-03) - PORTUGAL: 2♂, 1♀, Beira Alta, ca. 4km SSW of Manteigas, Serra da Estrela (Glacial Valley of the Zêzere), 40°22.158'N, 7°33.092'W, 1,109m, 17.v.2014, SW, leg. Marc Pollet & Anja De Braekeleer [MAPC] (W);

(PT-04) - PORTUGAL: 15♂, 8♀, Beira Alta, Serra da Estrela, 40°24'13''N, 7°35'10''W, 1,450m, 16-17.vii.2009, leg. Miroslav Barták [MIBC, see Naglis & Barták 2015] – labels: "PORTUGAL: Serra da Estrela, 40°24'13''N, 7°35'10''W, 1450 m, sweeping undergrowth of pine wood, 16–17.VII.2009";

(PT-05) - PORTUGAL: 1♀, Algarve, Ilha de Tavira, 37° 4'55.06"N, 7°41'1.12"W, 13.v.1989, leg. C.E. Dyte [BMNH] - labels: "PORTUGAL Algarve, Ilha" / "de Tavira, Saltmarsh" / "opposite Santa Luzia" / "13.v.1989 C.E. Dyte"; "BMNH(E) 2013-47" / " C.E. Dyte collection"; barcode "NHMUK010627337"; 1♀, same site, 13.v.1989, leg. C.E. Dyte [BMNH] - labels: "PORTUGAL Algarve, Ilha" / "de Tavira, Saltmarsh" / "opposite Santa Luzia" / "13.v.1989 C.E. Dyte"; "BMNH(E) 2013-47" / " C.E. Dyte collection"; barcode "NHMUK010627338";

(ES-01) - SPAIN: 1♂, Córdoba (Andalusía), Peñarroya, 1923-1924, leg. A. Seyrig [MNHN (collection générale)] - labels: "MUSEUM PARIS" / "ESPAGNE" / "PENARROYA" / "A. SEYRIG 1923-24"; "*Orthoceratium* ♂" / "*lacustre* (Scopoli)" / "H. Ulrich det. 1983";

(ES-02) - SPAIN: 4♀, Alicante (of auton. region Valencia), Elche, 7.v.1927, leg. G. Strobl [BMNH] - labels: "Elche 7/5 *Liancalus*" / "Südspanien lacustris" / "PROF. G. STROBL ♀"; [upside down] "Pres. by" / "E. Brunetti" / "B.M. 1927-184"; barcode "NHMUK010627344" [remarks: all specimens pinned on the same styrofoam]; 1♂, Alicante (of auton. region Valencia), Elche [NHMW] - labels: "Elche" / "Czerny" / [bottom side] "10/5 07"; 1♂, same locality [NHMW] - labels: "Elche" / "Czerny" / [bottom side] "10/5 07"; 1♂, same locality [NHMW] - labels: "Elche" / "Czerny"; 1♂, same locality [NHMW] - labels: "Elche" / "Czerny" / [bottom side] "9/5 07"; 1♂, same locality [NHMW] - labels: "Elche" / "Czerny" / [bottom side] "10/5 07"; 1♂, same locality [NHMW] - labels: "Elche" / "Czerny" / [bottom side] "10/5 07"; 1♂, same locality [NHMW] - labels: "Elche" / "Czerny" / [bottom side] "10/5 07"; 1♀, same locality [NHMW] - labels: "Elche" / "Czerny"; 1♂, same locality [NHMW] - labels: "Elche" / "Czerny" / [bottom side] "9/5 07"; 1♂, same locality [NHMW] - labels: "Elche" / "Czerny" / [bottom side] "10/5 07"; 1♀, same locality [NHMW] - labels: "Elche" / "Czerny" / [bottom side] "10/5 07"; 1♀, same locality [NHMW] - labels: "Elche" / "Czerny" / [bottom side] "10/5 07"; 1♂, same data - labels:

"Elche" / "Czerny" / [bottom side] "9/5 07"; 1♂, same locality [NHMW] - labels: "Elche" / "Czerny" / [bottom side] "10/5 07";

(**ES-03**) - **SPAIN**: 1♂, Segovia (of auton. region Castilla y Léon), Gudillos, 18.viii.1963, leg. A.O. Pont [BMNH] - labels: "swept around" / "stream near" / "GUDILLOS" / "c. 1400m"; "C. SPAIN" / "Sierra de Guadarrama" / "18.viii.1963" / "A.O.Pont"; "*Orthoceratium*" / "*lacustre* Scop." / "det. David Hollis, 1964"; barcode "NHMUK010627345"; 1♂, same location, 0♀, 18.viii.1963, leg. A.O. Pont [BMNH] - labels: "swept around" / "stream near" / "GUDILLOS" / "c. 1400m"; "C. SPAIN" / "Sierra de Guadarrama" / "18.viii.1963" / "A.O.Pont"; barcode "NHMUK010627346";

(**ES-04**) - **SPAIN**: 1♂, between Zaragoza (auton. region Aragón) y Soria (of auton. region Castilla y León), Moncayo, 29.vii.1921 [MNHN (collection générale)] - labels: "MONCAYO (I.)" / "29-VII-21"; "*Orthoceratium* ♂" / "*lacustre* (Scopoli)" / "H. Ulrich det. 1983";

(**ES-05**) - **SPAIN**: 1♀, Teruel (of auton. region Aragon), Noguera de Albarracín [NHMW] - labels: "Arag. Noguera" / "b. Albarracin" / "19.VII.'24 Zerny"; "*Orthoceratium*" / "*lacustre* Scop." / "det. O. Parent";

(**ES-06**) - **SPAIN**: 1♀, Cádiz (of auton. Region Andalusia), San Fernando [NHMW] - labels: "Hispania" / "J. Fernando" / "Abt Gerny" / [bottom side] "24/4 " / "07"; "*Lianc. lacustr*." / "♀ det. Strobl";

(**TN-01**) – see lectotype specimens; 1♀, Tunis (governorate), Tunis [MLUH] - labels: "Zool. Inst. Halle-S." / "*Orthoceratium*" / " *lacustre* Scop. ♀" / "det. O. Parent"; "v. Röder"; "TUNIS" / "AFRIKA.";

(**TN-02**) - **TUNISIA**: 1♀, Jendouba (governorate), Aïn Draham, 15.ix.1913 [ZMHB] - labels: "Tunisia" / "Ain Draham" / [bottom side] "1913" / "IX. 15."; "*lacustris*" / "Scop"; "Zool. Mus." / "Berlin";

(**TN-03**) - **TUNISIA**: 1♀, Ben Arous Governate, Hammam-Lif [NHMW] - labels: "Hammam Lif" / "Tunis Wagner '13";

(**TK-01**) - **TURKEY**: 1♀, inner Western Anatolia, Afyonkarahisar, Başmakçı, Akçapınar village 3rd km, 37°50′N, 29°59′E, 848m, 28.v.2009, SW, leg. Alper Tonguç [see Tonguç, Barlas & Grichanov, 2013];

(**TK-02**) - **TURKEY**: 1♂, 1♀, inner Western Anatolia, Afyonkarahisar, Başmakçı, Akçapınar village 6th km, 37°50′N, 29°58′E, 852m, 28.v.2009, SW, leg. Alper Tonguç [see Tonguç et al. 2013];

(**TK-03**) - **TURKEY**: 1♂, inner Western Anatolia, Centrum, Türkmen Mountain Türkmensu, 39°29′N, 30°19′E, 1,237m, 18.vii.2011, SW, leg. Alper Tonguç [see Tonguç et al. 2013];

(**TK-04**) - **TURKEY**: 1♀, inner Western Anatolia, Kütahya, Gediz,Küçükler-Murat mountain road 22nd km, 38°57′N, 29°36′E, 1,456m, 10.vi.2009, SW, leg. Alper Tonguç [see Tonguç et al. 2013];

(**TZ-01**) – **TANZANIA**: 1♂, Morogoro region, Kimboza Forest Reserve, 11.ix.1977, leg. Mahunka – labels: “Tanzania” / “Kimboza” / “Forest Reserve”; “11. ix. 1977” / “ leg. Mahunka”; “*Orthoceratium*” / “ *lacustre* Scop.” / “ det. Grichanov. 96”; [pale green] “HNHM-DIP_43”; “Orthoceratium sabulosum

(Becker, 1907) ♂” / “Det.: Marc Pollet” / “& Zoltán Soltész, 2018”; 1♀, Morogoro region, Matombo, 11.ix.1977, leg. Makunka – labels: “Tanzania” / “Matombo” / “Morogoro reg.”; “11. IX.1977.” / “leg. Mahunka”; “*Orthoceratium*” / “ *lacustre* Scop.” / “ det. Grichanov. 96”; “Orthoceratium sabulosum (Becker, 1907) ♀” / “Det.: Marc Pollet” / “& Zoltán Soltész, 2018”;

Two specimens of *Orthoceratium sabulosum* were labelled as follows: 1♀ [MNHN (collection générale)] - labels: "*Orthoceratium*" / "*lacustre* Scop ♀" / "O. Parent det."; 1♀ [MNHN (collection Parent)] - labels: "*Orth*. *Lacus*-" / "*tre* Scop".
